# Internal microdosimetry of alpha-emitting radionuclides

**DOI:** 10.1007/s00411-019-00826-w

**Published:** 2019-12-21

**Authors:** Werner Hofmann, Wei Bo Li, Werner Friedland, Brian W. Miller, Balázs Madas, Manuel Bardiès, Imre Balásházy

**Affiliations:** 1 Biological Physics, Department of Chemistry and Physics of Materials, University of Salzburg, Hellbrunner Str. 34, 5020 Salzburg, Austria; 2 Institute of Radiation Medicine, Helmholtz Zentrum München, Deutsches Forschungszentrum für Gesundheit und Umwelt (GmbH), Ingolstädter Landstr. 1, 85764 Neuherberg, Germany; 3 Department of Radiation Oncology, School of Medicine, University of Colorado, Aurora, CO 80045 USA; 4 College of Optical Sciences, University of Arizona, Tucson, AZ 85721 USA; 5 Environmental Physics Department, MTA Centre for Energy Research, Budapest, Hungary; 6 Centre de Recherches en Cancérologie de Toulouse, UMR 1037, INSERM Université Paul Sabatier, Toulouse, France

**Keywords:** Internal dosimetry, Microdosimetry, Alpha-emitting radionuclides

## Abstract

At the tissue level, energy deposition in cells is determined by the microdistribution of alpha-emitting radionuclides in relation to sensitive target cells. Furthermore, the highly localized energy deposition of alpha particle tracks and the limited range of alpha particles in tissue produce a highly inhomogeneous energy deposition in traversed cell nuclei. Thus, energy deposition in cell nuclei in a given tissue is characterized by the probability of alpha particle hits and, in the case of a hit, by the energy deposited there. In classical microdosimetry, the randomness of energy deposition in cellular sites is described by a stochastic quantity, the specific energy, which approximates the macroscopic dose for a sufficiently large number of energy deposition events. Typical examples of the alpha-emitting radionuclides in internal microdosimetry are radon progeny and plutonium in the lungs, plutonium and americium in bones, and radium in targeted radionuclide therapy. Several microdosimetric approaches have been proposed to relate specific energy distributions to radiobiological effects, such as hit-related concepts, LET and track length-based models, effect-specific interpretations of specific energy distributions, such as the dual radiation action theory or the hit-size effectiveness function, and finally track structure models. Since microdosimetry characterizes only the initial step of energy deposition, microdosimetric concepts are most successful in exposure situations where biological effects are dominated by energy deposition, but not by subsequently operating biological mechanisms. Indeed, the simulation of the combined action of physical and biological factors may eventually require the application of track structure models at the nanometer scale.

## Introduction

The absorbed dose is commonly regarded as the relevant physical quantity to relate the exposure to ionizing radiation to epidemiologically or pathologically observed health effects, such as cancer. Absorbed dose, *D*, is defined as the quotient of ${\text{d}}\bar{\varepsilon }$ by dm (ICRU 1980), where ${\text{d}}\bar{\varepsilon }$ is the mean energy imparted by ionizing radiation to matter of mass dm, i.e.,

1 $$ D = {\text{d}}\bar{\varepsilon }/{\text{dm}} $$

The unit of absorbed dose is J kg^−1^ and the special name for that unit is gray (Gy). Note that absorbed dose *D* is considered a point quantity, but it should be recognized that the physical process does not allow dm to approach zero in the mathematical sense. Thus, for practical dose calculations, dm often refers to a 1 µm unit density sphere.

Macroscopic dosimetry, or dosimetry at the organ level, refers to the dosimetry in macroscopic biological targets, such as the organs of the human body or specific tissues in a given organ. For example, the lung is the primary general target for the inhalation of radionuclides, while the bronchial epithelium is the specific target for bronchial carcinomas arising from the inhalation of radon progeny. Since d$\bar{\varepsilon }$ is the mean energy imparted to a macroscopic volume of mass dm, dose *D* is a deterministic quantity.

Incorporated alpha-emitting radionuclides represent a special case in internal dosimetry. Due to the highly localized energy deposition of alpha particles along short, straight tracks, energy deposition in cells or cell nuclei in an irradiated organ or tissue will be highly inhomogeneous. Note that cellular radiobiological effects depend on the energy actually deposited in a given cell and not on a hypothetical mean value over all cells in a given irradiated tissue volume. Thus, microdosimetry, or dosimetry at the cellular level, refers to the dosimetry in sensitive target cells or, more specifically, in their cell nuclei as the primary target site for cellular radiobiological effects relevant for carcinogenesis, such as oncogenic transformation or cell killing.

A specific peculiarity of internal microdosimetry is the spatial variability of the target distribution within a given tissue volume and the spatial variability of the radionuclide distribution emitting alpha particles. For example, basal and secretory cells in bronchial epithelium are located at variable depths in bronchial epithelium and their relative frequencies vary with their location in the bronchial region (Mercer et al. 1991). Moreover, incorporated alpha-emitting radionuclides are usually non-uniformly distributed within an organ or tissue, such as radon progeny accumulations at bronchial airway bifurcations (Hofmann et al. 2000a; Balásházy and Hofmann 2000; Fakir et al. 2005b). Due to the limited range of alpha particles, a strong geometric relationship exists between the emission sites of alpha particles and the cellular target sites. Thus, internal microdosimetry is characterized by the superposition of two distributions, the microdistribution of alpha-emitting radionuclides in an organ or tissue and the microdistribution of target cells in a tissue. Consideration of the spatial correlation of source and target distributions yields distribution of mean cellular doses, where cellular doses are either determined as localized point doses or by assuming mean cellular chord lengths and an average linear energy transfer (LET). Although such calculations are performed at the microscopic scale, and thus represent a first step to cellular microdosimetry, this approach is still based on the macroscopic dose concept.

Therefore, the next step in internal microdosimetry is the consideration of the randomness of energy deposition within cells or cell nuclei. Termed classical microdosimetry, the dosimetric equivalent unit of the absorbed dose at the cellular level is the specific energy *z* (ICRU 1983; Li et al. 2018; Rossi 1968; Kellerer and Chmelevsky 1975a, b, c; Rossi and Zaider 1996), defined in analogy to the absorbed dose as the quotient of d*ε* by dm, except that d*ε* is the actual energy imparted to mass element dm. Since microdosimetry considers the randomness of energy deposition in cellular sites, the specific energy is a stochastic quantity. However, for a sufficiently large number of energy deposition events, i.e., for medium to high doses, the average specific energy approximates the macroscopic dose *D*.

Since alpha particle tracks in tissue are represented by straight lines, cellular microdosimetric calculations are primarily a geometric problem. Thus, the randomness of energy deposition events in cell nuclei depends on (1) the probability of hitting the target due to the limited range of alpha particles as a result of the spatial correlation between emission site and target site, (2) the random intersection lengths of alpha particles in target cell nuclei (crossers) or incomplete traversal (stoppers), (3) the random energy deposition along a given track as a result of the variable stopping power (Bragg curve), and, (4) the frequency of single and multiple hits as a result of local accumulations of the alpha-emitting radionuclide activity in a given tissue.

Since radiobiological effects observed at the cellular level, such as oncogenic transformation, originate at the DNA level, the tools of classical microdosimetry, such as specific energy distributions, are too coarse for the interaction of ionizing radiation with DNA targets and thus have to be supplemented by the track structure approach. Hence, nanodosimetry, or dosimetry at the DNA level for alpha particles describes the interaction of particle tracks with DNA components, such as chromosomes (Friedland et al. 2011; Friedland and Kundrát 2013; Li et al. 2018; Paretzke 1987; Zaider and Varma 1992).

**Fig. 1:**
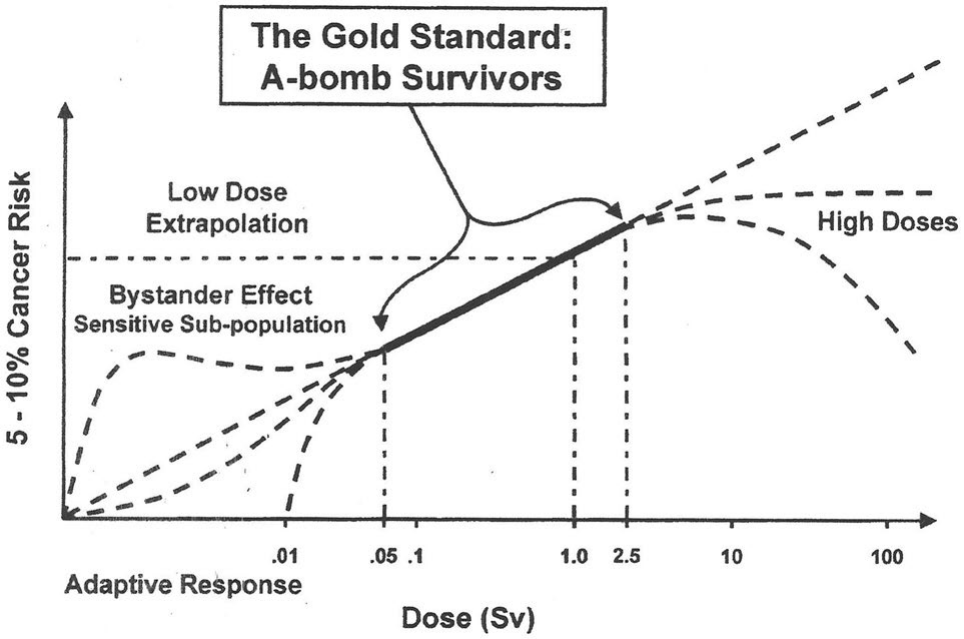
Dose–response relationship for radiation-induced carcinogenesis. Reprinted from Hall (2004) with permission of Taylor & Francis

The microdosimetric approach is especially relevant for the dose–response relationship at low doses and dose rates. This is illustrated in Fig. 1 for radiation-induced carcinogenesis in humans (Hall 2004). Below about 50 mSv, the statistical uncertainties of the epidemiological data do not allow an unequivocal determination of the actual dose–effect curve. For reasons of ethics and practicability, international radiation protection organizations, such as the International Commission on Radiological Protection (ICRP 2007), recommend a linear extrapolation from the higher dose data down to the low dose region. However, bystander mechanisms and the existence of sensitive subpopulations suggest that the linear extrapolation would underestimate the actual risk, while adaptive response suggest that it overestimates the low dose risk. Thus, the epidemiological uncertainties and the uncertainty of operating non-linear cellular mechanisms require a detailed physical description of the energy deposition at the cellular or DNA level.

The microdosimetric description is most relevant for low level alpha particle exposures, where low doses of alpha particles are characterized by a small number of cells affected, which may receive relatively high cellular doses, while the majority of cells are not hit at all. At low doses and dose rates, such as low level radon exposures, isolated energy deposition events in a cellular matrix occur over an extended period of time. Hence, the average energy deposition in cells in a given tissue is a somewhat meaningless quantity to predict potentially carcinogenic radiobiological effects in cells.

Starting with the principles of internal microdosimetry (“Principles of internal microdosimetry”), the present paper on the internal microdosimetry of alpha-emitting radionuclides reviews the currently available studies on alpha microdosimetry calculations in the human lung (“Alpha particle microdosimetry in the human lungs”), in bones (“Alpha particle microdosimetry in bones”) and in targeted radionuclide therapy (“Alpha particle microdosimetry in targeted radionuclide therapy”). This is followed by the analyses of the radiobiological implications of microdosimetry, such as the extrapolation of radiobiological effects from the DNA level (nanodosimetry) to the cellular level (microdosimetry) (“Alpha particle tracks in silico: from nanoscale to microscale”), the microdosimetric interpretation of in vitro cellular radiobiological effects (“Relationship between microdosimetry and cellular radiobiological effects”), and the relevance of cellular microdosimetry for tissue and organ dosimetry (“From cellular microdosimetry to the tissue and organ level”). Finally, the advantages and limitations of the microdosimetric approach for alpha particles will be discussed (“Applicability and limitations of alpha particle microdosimetry”), including practical implications for radiation protection, radiobiology and nuclear medicine.

## Principles of internal microdosimetry

### Microdosimetric quantities

The definitions of the microdosimetric quantities and distributions can be found in the International Commission on Radiation Units and Measurements (ICRU) report on microdosimetry (ICRU 1983). However, for the sake of the reader, the most relevant quantities will be repeated here. The two basic stochastic quantities describing the microscopic distribution of energy deposition in microscopic targets are the specific energy, *z*, and the lineal energy, *y*.

In analogy to the definition of the macroscopic quantity absorbed dose, *D*, the specific energy *z* (also sometimes called event size) is defined as the quotient of *ε* by *m*, where *ε* is the energy imparted by ionizing radiation to matter of mass *m*, i.e.,

2 $$ z = \frac{\varepsilon }{m} $$

The unit of *z* is J kg^−1^ and the special name for that unit is gray (Gy). Since *z* is a stochastic quantity, it can be characterized by the distribution function *F*(*z*). The probability density, *f*(*z*), is then defined as the derivative of *F*(*z*):

3 $$ f\left( z \right) = {\text{d}}F\left( z \right)/{\text{d}}z $$

The probability density, *f*(*z*), includes a discrete component, the delta function^1^, *δ*, at *z* = 0 for the probability of no energy deposition, which also represents the fraction of unirradiated targets. Hence, the mean number of hits *M* = − ln*δ*.

The expectation value of the specific energy, which is a non-stochastic quantity,

4 $$ \bar{z} = \mathop \int \limits_{0}^{\infty } zf\left( z \right){\text{d}}z $$

is called mean specific energy.

The specific energy may be due to one or more energy deposition events. Thus, the probability density *f*_1_(*z*) of the specific energy deposited in a single event, *F*_1_(*z*), is called the single-event distribution of *z*. The expectation value of *f*_1_(*z*), $\bar{z}_{1}$ or $\bar{z}_{\text{F}}$, called frequency-mean specific energy per event, is a non-stochastic quantity. The number, *n*, of energy deposition events which have contributed to a particular specific energy, *z*, is, in general, distributed at random and is commonly described by a Poisson distribution.

**Fig. 2:**
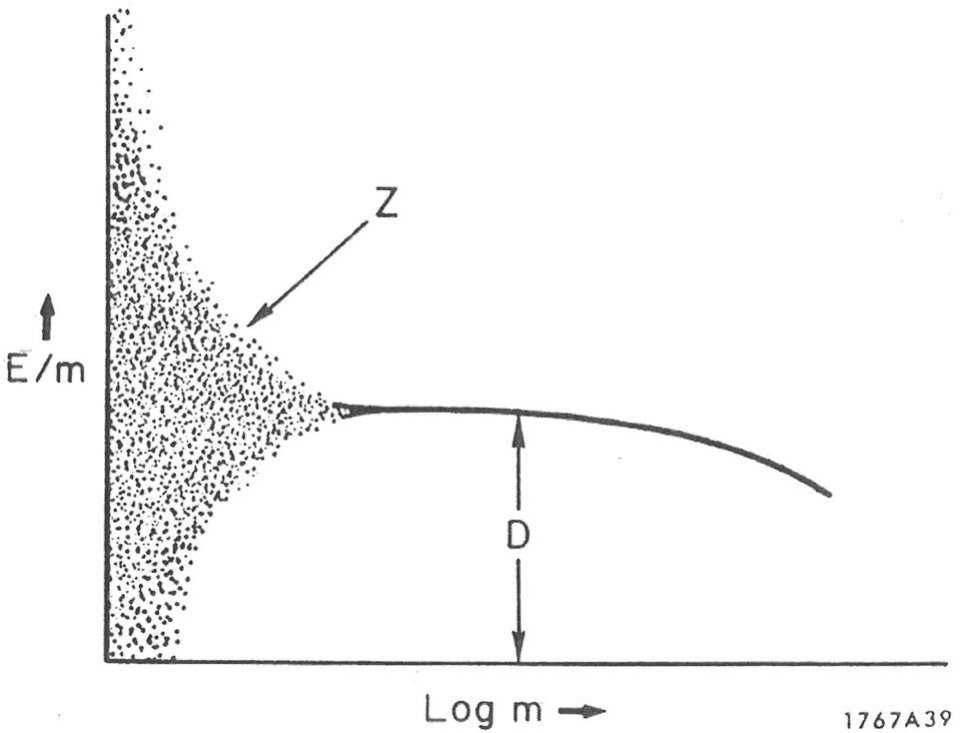
Variation of the specific energy with size (mass) of a microscopic target. Reprinted from Rossi (1968) with permission of Elsevier

The variation of the specific energy *z* with mass *m* of a microscopic target is illustrated in Fig. 2 (Rossi 1968). For very small masses (or volumes) of micrometer-sized targets, such as cell nuclei or DNA components, the specific energy can vary over a wide range of values. For sufficiently large masses or multiple energy deposition events, *f*(*z*,*D*), the specific energy approaches a mean value $\bar{z}$, which is equivalent to the absorbed dose *D*:

5 $$ \bar{z} = \mathop \int \limits_{0}^{\infty } z f\left( {z;D} \right) {\text{d}}z = D $$

This figure clearly demonstrates that absorbed dose *D* is an inappropriate quantity to describe the randomness of energy deposition in microscopic targets and at low doses. In analogy, the macroscopic quantity linear energy transfer, LET, the lineal energy or event size, *y*, is the quotient of *ε* by $\bar{l}$, where *ε* is the energy imparted to the matter in a volume by a single energy deposition event and $\bar{l}$ is the mean chord length in that volume:

6 $$ y = \varepsilon_{\text{S}} /\bar{l} $$

The unit of lineal energy *y* is J m^−1^, but most commonly, the unit used for this quantity is keV µm^−1^.

The mean chord length $\bar{l}$ in a volume is the mean length of randomly oriented chords in that volume. For a convex body, $\bar{l}$  = 4 *V*/*A*, where *V* is the volume and *A* is the surface area of this body (note: for a spherical volume with radius *r*, $\bar{l}$  = 4/3 *r*). Since *y* is a stochastic quantity, it can be characterized by the distribution function *F*(*y*). The probability density, *f*(*y*), is then defined as the derivative of *F*(*y*):

7 $$ f\left( y \right) = {\text{d}}F\left( y \right)/{\text{d}}y $$

It is important to point out that *y* is defined for single energy deposition events only. Thus, the linear energy distribution, *f*(*y*), is independent of the absorbed dose or dose rate. Its expectation value is again a non-stochastic quantity. The probability densities of *z* and *y*, i.e. *f*(*z*) and *f*(*y*), were first determined experimentally by Rossi (1968) for different types of ionizing radiation with proportional counters.

### Microdosimetry of internal alpha-emitters

Roesch (1977) extended the fundamental concepts of external microdosimetry to internally deposited radionuclide sources, with special application to ^239^Pu alpha particles. The computational procedure is as follows (Li and Zheng 1996): first, the probability density function in specific energy due to single energy deposition events, $\bar{z}_{1}$, is determined. For a given absorbed dose *D*, the mean number of energy deposition events, *N*, is *D*/$\bar{z}_{1}$. The resulting specific energy probability density for exactly *n* single events, *f*_*n*_(*z*), can be calculated as the n-fold convolution of the single-event density *f*_1_(*z*) using the Fourier transform technique:

8 $$ f_{n} (z) = \int_{0}^{z} {f_{1} (z)f_{n - 1} (z - z^{{\prime }} )} {\text{d}}z^{{\prime }} $$

Since the probability of exactly *n* energy deposition events at absorbed dose *D* follows the Poisson distribution, the dose-dependent specific energy distribution, *f* (*z*;*D*), is given by the compound Poisson process:

9 $$ f\left( {z;D} \right) = \mathop \sum \limits_{n = 0}^{\infty } \frac{{M^{n} }}{n!}e^{ - M} \left[ {f_{1} \left( z \right)} \right]^{*n} $$

In the case of internal radionuclides, the alpha activity is represented by a distribution of point sources with multiple emissions, where *f*_1_(*r*,*z*) is the single-event density for a point source as a function of distance *r* from the target site. If the target receives energy deposition events from several point sources, the probability density in specific energy in that target is the convolution of the specific energy densities contributed by each point source independently. Since the probability of exactly *m* point sources hitting the target site *n* times follows a Poisson distribution, the resulting specific energy distribution is again described by a compound Poisson process.

As an alternative approach to the analytic code of Roesch (1977), Aubineau-Laniece et al. (2002) and Fakir et al. (Fakir et al. 2005a; Fakir et al. 2005b, 2006) developed a stochastic approach for the special case of radon progeny alpha particles emitted from cylindrical bronchial airway surfaces. The random emission of alpha particle tracks, their intersection with cell nuclei and the resulting energy deposition were simulated by Monte Carlo methods. Multi-event distributions in epithelial cells of bronchiolar airways calculated by both analytic and Monte Carlo methods revealed excellent agreement between the two codes (Aubineau-Laniece et al. 2002).

**Fig. 3:**
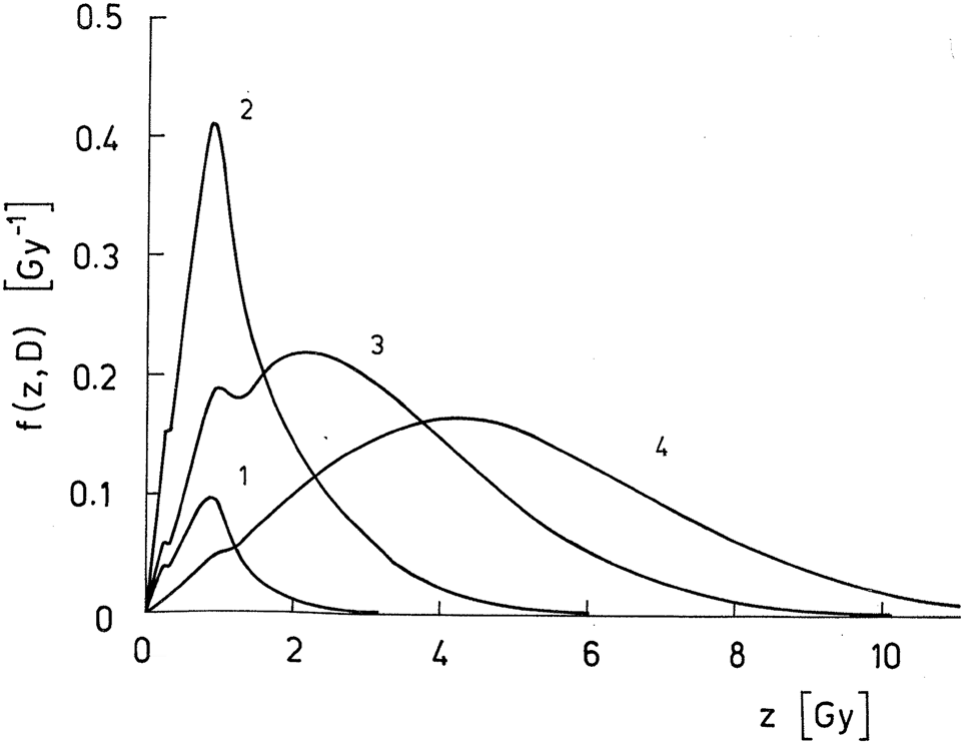
Specific energy distributions *f*(*z*;*D*) for basal cell nuclei calculated for four values of the absorbed dose *D* for specific radon progeny exposure conditions. The corresponding fraction of cells nuclei missed by alpha particles is given by the delta function *δ*. Curve 1: *D* = 0.1 Gy, *δ* = 0.90; curve 2: *D* = 1 Gy, *δ* = 0.35; curve 3: *D* = 3 Gy, *δ* = 0.043; curve 4: *D* = 5 Gy, δ = 0.0053 (Sedlák 1996). Reprinted from Sedlák (1996) with permission of Wolters Kluwer Health, Inc

To illustrate the calculation of *f*(*z*;*D*), Fig. 3 shows specific energy distributions for basal cell nuclei for absorbed doses ranging from 0.1 to 5 Gy for specific radon progeny exposure conditions (Sedlák 1996). Curves 1 and 2 represent energy deposition by single alpha particle hits, whereas the increasing number of multiple alpha particle hits at higher doses shifts the specific energy distributions to higher *z* values. While the *f*(*z*;*D*) distribution for the highest dose of 5 Gy is nearly symmetrical around the absorbed dose *D* (curve 4), the specific energy distributions for the smaller doses are skewed towards smaller *z* values with an extended high-energy tail (curves 1–3 for *D* = 0.1, 1, and 3 Gy, respectively). The areas under curves correspond to the fraction of nuclear hits, ranging from 10% for *D* = 0.1 Gy to 9.9947 for *D* = 5 Gy. Corresponding specific energy distributions for secretory cells are very similar to the distribution for the basal cells shown in Fig. 3 except that the fraction of cellular hits is slightly higher as they are located closer to the emission sites.

Internal alpha particle microdosimetry at the cellular levels, i.e., the variability of energy deposition in cells or cell nuclei, encompasses problems of internal dosimetry and cellular microdosimetry. Specific internal dosimetry aspects are (1) the inter- and intrasubject variability of radionuclide deposition in a given source organ, (2) the microdistribution of radionuclides within a given target tissue, and (3) the spatial correlation between the microdistribution of radionuclides and the target cell distribution resulting from the limited range of alpha particles. Examples of non-uniform nuclide and target distributions are (1): short-lived alpha-emitters (radon progeny) in the bronchial region of the lung, (2) long-lived alpha-emitters (Pu) in the alveolar region of the lung, and, (3) long-lived alpha-emitters in bones (Ra, Pu).

Specific cellular microdosimetry parameters are (1) the probability of hitting the target due to the limited range of alpha particles, and (2) in the case of a hit, the variability of energy deposition in cells or cell nuclei, such as the number of cellular hits (single or multiple hits), the randomness of track intersection lengths in the target volume by traversing alpha particles (crossers and stoppers), and, finally, the LET dependence of the intersecting track as a result of the spatial correlation between emission site and target site (Bragg curve). In the case of alpha particles and micrometer-sized cellular targets, e.g., cell nuclei, range and energy straggling as well as the contribution of *δ*-rays generated outside the target may be neglected. In conclusion, cellular microdosimetry of alpha particles can be characterized by two dosimetric parameters, the number of cellular hits and the specific energy distribution for single and multiple hits, expressed by *f*(*z*;*D*).

The significance of the hit probability can be demonstrated by the analysis of the extrapolation from high to very low doses in terms of the number of alpha particle hits in a cellular volume and the number of cells hit in the tissue by an alpha particle. At sufficiently high average doses in a group of cells, each cell is hit at least a few times (hit probability = 1, multiple hits). With decreasing average dose, not all cells are hit anymore and cells hit may receive one or more hits (hit probability < 1, single and multiple hits). Finally, very low average doses are characterized by single alpha particle hits in only very few cells (hit probability ≪ 1, single hits). However, due to the high energy deposited by a single alpha particle, the cells hit receive a relatively high cellular dose. Thus, a very low average tissue dose produced by alpha particles is an indicator of a small number of cells hit, rather than a small cellular dose. In other words, a low average tissue dose is a substitute for the average fraction of the number of cells affected in a tissue volume and not of the average cellular dose. Thus with increasing average dose, the number of cell hit increases but not the cellular dose, until multiple hits start to play a role.

While classical microdosimetry is an extension of the dose concept, i.e., deposition of energy in a given volume, at the microscopic level, a different approach has been proposed by Katz (1987). In his effect-specific track structure model, energy deposition along a charged particle track and resulting cellular biological effects are related to the path of a single particle, where the energy deposition around the path of a charged particle track is described by the radial dose distribution produced by secondary electrons, averaged over the sensitive site at a given distance from the path of the particle. The link between the radial dose distribution and the resulting biological response in irradiated cells, based on the single-hit, multi-target formalism, is determined by four radiosensitivity parameters, which can be obtained from effect-specific radiobiological in vitro studies in which cells are irradiated with gamma rays and different heavy ions exhibiting a wide range of LET values. The application of this model requires the availability of pertinent radiobiological data, hence the term “effect-specific”. This model was specifically developed for external irradiation with heavy charged particles, but it is also applicable to internal alpha particles (Hofmann et al. 1996b). Since macroscopically averaged gamma ray dose is used in this model as its reference radiation, it cannot be applied to gamma radiation. This phenomenological track structure model can be considered as a first step towards the simulation of energy deposition at the nanometer scale by current track structure codes described later in the text.

## Alpha particle microdosimetry in the human lungs

Examples of microdosimetric calculations for alpha particles in the human lung published in the past are short-lived alpha-emitters, e.g., radon progeny, in the bronchial region of the lung and long-lived alpha-emitters, e.g., plutonium, in the alveolar region of the lung. In the case of radon progeny, alpha-emitting nuclides ^218^Po and ^214^Po on bronchial airway surfaces represent a cylindrical surface source and sensitive target cells are located at given depths in the surrounding bronchial epithelium. Thus, due to the short ranges of alpha particles in tissue, a strong geometric correlation exists between alpha particle sources and target sites. In contrast, long-lived alpha-emitters in the alveolar region represent randomly located particulate sources and target cells are randomly distributed throughout the alveolar tissue. Together with the longer ranges of alpha particles due to the lower tissue density, there is a random correlation between alpha sources and target sites.

### Microdosimetry of radon progeny alpha particles in bronchial target cells

The short-lived radon progeny emitting alpha particles are ^218^Po (half-life = 3.07 min, *E*_α_ = 6.11 MeV) and ^214^Po (half-life = 162 µs, *E*_α_ = 7.83 MeV). Their ranges in soft tissue are approximately 47 µm and 71 µm, respectively. Basal and secretory cells are currently considered as the sensitive target cells in the bronchial epithelium as potential progenitor cells of bronchial carcinomas (ICRP 1994; ICRU 2015). The nuclei of these target cells can be approximated by spheres with average diameters of about 10 µm (Mercer et al. 1994). Current radon lung dosimetry models are generally based on the absorbed dose concept, permitting the calculation of cellular doses to both target cells in different bronchial airway generations (Hofmann and Winkler-Heil 2011). The steady-state surface activities of both radon progeny nuclides on bronchial airway surfaces are produced by the initial deposition of inhaled radon progeny and their subsequent clearance by mucociliary action. Alpha particles emitted from the airway surfaces can hit the basal and secretory cells in the bronchial epithelium, provided that the distance between the emission site and the target site is within the range of the alpha particles.

Two macroscopic sources of randomness contribute to the microscopic energy deposition in the nuclei of sensitive target cells: (1) the inter- and intrasubject variability of radon progeny surface activities in a given airway generation, and, (2) the depth distribution of target cells and their relative frequencies within the epithelial tissue affecting the frequency of cellular alpha particle hits (Hofmann and Daschil 1986; Hofmann et al. 1996c, 2010). As a result of the inherent variability of bronchial airway dimensions, the resulting variability of deposition fractions and mucociliary clearance velocities leads to a wide distribution of ^218^Po and ^214^Po surface activities (Hofmann et al. 2000a). This source variability is further enhanced by the variable thickness of the bronchial epithelium, the depth distribution of target cells within epithelial tissue and their relative frequencies. The spatial correlation between the microdistribution of radionuclides and the target cell distribution and the limited range of alpha particles affects the probability and multiplicity of cellular hits and the energy deposition as a function of the alpha particle range (Bragg curve). The resulting cellular dose distributions can be approximated by lognormal distributions (Hofmann and Daschil 1986; Hofmann et al. 2000a).

While these cellular dose distributions are based on uniform nuclide distributions on bronchial airway surfaces within a given airway generation, experimental (Kinsara et al. 1995) and CFD simulation studies (Balásházy and Hofmann 2000, 1999; Balásházy et al. 2002; Hofmann et al. 1996a) for inhaled attached and unattached radon progeny in bronchial airway bifurcation models have demonstrated that inhaled nuclides are preferentially deposited within bifurcation zones, thereby producing radionuclide hot spots in the vicinity of the peak of the bifurcation (carinal ridge). This localized deposition is further enhanced by the reduced mucociliary clearance at airway branching sites (Hofmann et al. 1990a). This inhomogeneous source distribution further increases the randomness of cellular doses.

Microscopic sources of the energy deposition in microscopic targets are (1) the random track lengths of alpha particles, either traversing the target (crossers) or stopping within the target at the end of the alpha particle range (stoppers), (2) the random energy deposition along its path as a result of the LET as a function of the alpha particle range (Bragg curve), and (3) the probability of single and multiple hits following a Poisson distribution.

**Fig. 4:**
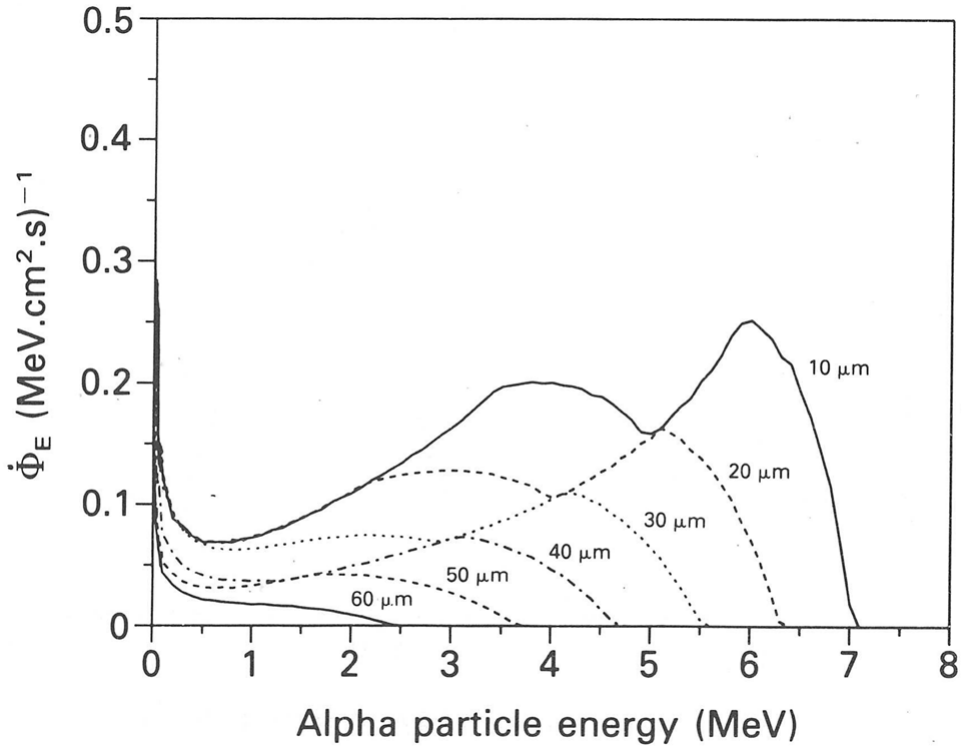
Energy or fluence rate spectra, Φ_E_, for ^214^Po alpha particles at various cell depths in bronchial epithelium of airway generation 2, normalized to a ^214^Po surface activity of 1 Bq cm^−2^. Reprinted from Caswell et al. (1994) with permission of Oxford University Press

Energy deposition in cell nuclei located at a given depth in bronchial epithelium depends on the energy of the alpha particles intersecting these cells. Due to variable distances between the uniform distribution of emission sites on bronchial airway surfaces and the target cells, the alpha particle energies exhibit a wide range of energies for a given exit energy. Since the energy spectrum of alpha particles in a given cell nucleus determines the stopping power and the LET, the determination of slowing down energy spectra is the starting point of all subsequent calculations of average cellular doses and microdosimetric specific energy and lineal energy distributions (Fig. 4) (Caswell and Coyne 1990a, b; Caswell et al. 1994; Fakir et al. 2005a, b, 2008). The steady decrease of the alpha particle fluence rate and the related maximum energy with cell depth is caused by the ever increasing attenuation of alpha particles due to their longer travel distances in tissue.

While still based on the absorbed dose concept, hit probabilities represent a more accurate measure of the probability of a cell nucleus sustaining damage following irradiation by alpha particles than tissue dose (Crawford-Brown and Shyr 1987). For alpha-emitting radon progeny activities on bronchial airways surface, computed hit probabilities for single and multiple hits in sensitive bronchial target cells, assuming a Poisson distribution, combined with average cellular doses, provide a more realistic basis for resulting cellular radiobiological effects than an average tissue dose. For example, calculations of frequencies of single and multiple cellular alpha particle hits in bronchial target cells have been published by Balásházy et al. (2009), Crawford-Brown (1988), Fakir et al. (2005b), Farkas et al. (2011), Harley (1988), Hofmann et al. (1994, 2002), Hui et al. (1990), Madas (2016), Nikezic and Yu (2001), Sedlák (1996), Szoke et al. (2006, 2009, 2012) and Truta-Popa et al. (2011a). Important findings from a dosimetric point of view are: (1) hit probabilities decrease in a nearly linear manner with increasing depth in bronchial tissue, (2) for typical indoor radon progeny activity concentrations, only few target cells are hit during the lifetime of these cells and multiple hits will play a role only for cells located at airway bifurcations due to the localized radon progeny accumulations, and, (3) the frequency of cellular hits increases with rising radon progeny activities from low domestic to high uranium miner exposures where multiple hits have to be considered.

Since experimentally determined radiobiological effects, such as cell killing, mutation and transformation are commonly expressed in terms of cellular doses, hit probabilities combined with average cellular doses are sometimes used to relate energy deposition at the cellular level to cellular radiobiological effects within lung tissue (Balásházy et al. 2009; Hofmann et al. 1990b, 1994; Madas et al. 2011; Szőke et al. 2006, 2009, 2012; Truta-Popa et al. 2011a). Although such simulations do not consider fluctuations of energy deposition within cells or cell nuclei, they are sometimes referred to as microdosimetric calculations.

While hit probabilities combined with average cellular doses capture the randomness and inhomogeneity of source and target distributions, they do not consider the randomness of energy deposition within a traversed target cell. Fortunately, in the case of alpha particles, energy deposition in cellular targets is determined by the intersection of straight lines with spherical targets and the energy deposited in a single traversal is proportional to the track length within the target, exhibiting a triangular shape. For example, track length distributions of radon progeny alpha particles in bronchial epithelial cells were computed by Hofmann et al. (2000a), Szőke et al. (2009) and Farkas et al. (2011), considering the energy spectra of alpha particles incident upon the cell nuclei at a given depth.

Although the calculation of track length distributions within cells or cell nuclei for single traversals, together with the multiplicity of traversals, already represents a first step to consider the stochastic aspects of cellular energy deposition, it is the concept of specific energy distributions which provides a complete description of the randomness of energy deposition in micrometer sites, containing information on both microscopic energy deposition and hit probabilities. For example, calculations of specific energy distributions in bronchial target cells by radon progeny alpha particles have been reported (Al-Affan 1994; Aubineau-Laniece et al. 2002; Fakir et al. 2005a, b, 2008; Fisher et al. 1991, 1992; Hofmann 1982, 1983a, 1984, 2007; Hui et al. 1990; Li and Zheng 1996; Nikezic and Yu 2002a, b, 2003).

For low bronchial tissue doses, energy deposition in single cells or cell nuclei is caused by the action of single alpha particle hits and is hence characterized by the single-event distribution *f*_1_(z). For example, single-event specific energy distributions *f*_1_(*z*) of ^218^Po and ^214^Po alpha particles in basal and secretory cell nuclei in the bronchial epithelium of the human lung for a cumulative exposure of 0.023 WLM^2^ during the average lifetime of an epithelial cell of 30 days are practically identical (Hui et al. 1990), except for their maximum values. The slightly lower peak of the specific energy distribution for the basal cells reflects the distal position of basal cell nuclei in bronchial epithelium as compared to the shallow lying secretory cells, and hence smaller hit probabilities.

In the case of target cells in bronchial airway bifurcations, local accumulations of the radon progeny at the carinal ridge can produce multiple hits in these target cells even at low tissue doses (Hofmann et al. 1990a). For example, Fakir et al. (2005b) computed specific energy spectra for ^218^Po and ^214^Po alpha particles in bronchial secretory and basal cell nuclei for three different target cell locations in an asymmetric bronchial airway bifurcation, corresponding to bronchial airway generations 3 and 4, for a cumulative lifetime exposure of 20 WLM, which is characteristic for residential radon exposures. These three target cell locations ranged from (1) the carinal ridge with a localized enhanced activity (hot spot) (T), to (2) the cylindrical part of the bifurcation with a uniform average activity (*R*_2_), and (3) the intermediate curved transition zone which receives alpha particle hits from both the hot spot and the uniform activity distribution (*R*_1_). While the specific energy spectra in *R*_1_ and *R*_2_ both represent single-event distributions, the specific energy spectrum in *T* demonstrates the effect of multiple alpha particle intersections.

**Fig. 5:**
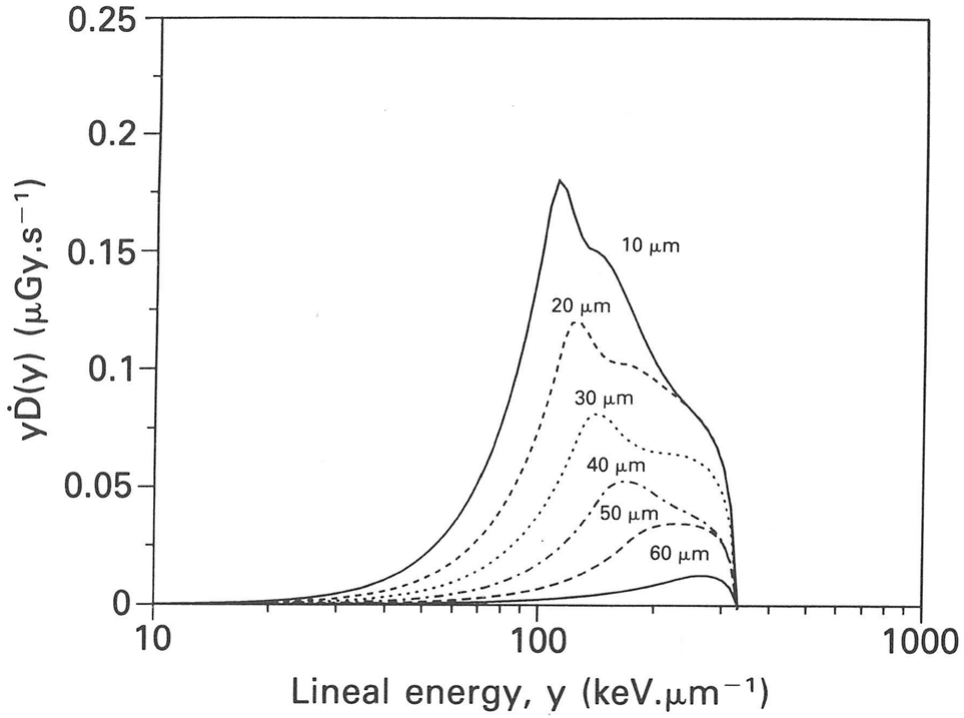
Lineal energy spectra for ^214^Po alpha particles at various cell depths in epithelial tissue in bronchial airway generation 2, normalized to a ^214^Po surface activity of 1 Bq cm^−2^. Reprinted from Caswell et al. (1994) with permission by Oxford University Press

In addition to specific energy spectra, *f*(*z*), corresponding lineal energy spectra, *f*(*y*), of radon progeny alpha particles in bronchial epithelium have been calculated by several authors (Brenner 1990; Caswell and Coyne 1990a, b; Caswell et al. 1994; Fakir et al. 2005a, 2008; Hofmann et al. 1994; Nikezic and Yu 2002a; Zaider and Varma 1992). For example, lineal energy spectra for ^214^Po alpha particles at various cell depths in epithelial tissue in bronchial airway generation 2 are plotted in Fig. 5 (Caswell et al. 1994). Note that the areas under the curves correspond to dose rate, which steadily decreases with cell depth. Consistent with the lower alpha particle energies at greater depths, and therefore higher stopping powers, the maximum of the lineal energy spectrum is shifted to higher *y*-values.

### Microdosimetry of long-lived alpha-emitting radionuclides in alveolar target cells

In the 1970s, the hot particle controversy, i.e., whether plutonium hot particles were more carcinogenic than uniformly distributed alpha-emitting particles of the same activity, uncovered the limitations of an average tissue dose for the prediction of health effects (EPA 1976; NCRP 1975). Alpha particle energies for ^238^Pu are 5.55 MeV (29%) and 5.59 MeV (71%) and 5.19 MeV (13%), 5.23 MeV (17%) and 5.24 Mev (70%) for ^239^Pu, respectively.

In analogy to the calculation of hit probabilities of radon progeny alpha particles in bronchial airways (Crawford-Brown and Shyr 1987), Kellerer (1978) derived equations for the frequency of alpha particles emitted from ^239^PuO_2_ in cells in the alveolar region of the lung for the National Council on Radiation Protection and Measurements report (NCRP 1975). If a given ^239^PuO_2_ activity is uniformly dispersed in the lungs, then a maximum number of cells is traversed by alpha particles and all cells have the same probability. However, the probability of multiple traversals of one cell, resulting from the Poisson distribution, is comparatively small. If, on the other hand, the activity is contained in ^239^PuO_2_ particles, then the probabilities for single and multiple traversals depend on the distance of a given cell from the particle. Hence, cells close to these particles will be subject to multiple traversals and the total number of cells intersected will be accordingly smaller.

In contrast to radon progeny in bronchial airways, where a strong geometric correlation exists between the cylindrical alpha particle source and cellular target sites surrounding the airways, target cells are randomly distributed throughout the air-filled parenchymal tissue. Due to the generally long radioactive half-lives and clearance times, nuclide distributions may change over time. For example, microdistributions of the activities of long-lived alpha-emitting nuclides of the natural uranium and thorium decay series in human lung tissue have been measured by Henshaw and Fews (1983, 1984) and Henshaw et al. (1988) with nuclear track detectors. The random distribution of air and tissue within a given lung volume leads to random intersection lengths in air and tissue and hence to highly variable ranges of alpha particles. Thus, the distribution of distances between alpha emission sites and target cells is a major determinant of local dose distributions.

Based on the nuclide microdistributions, two different computational methods have been employed to simulate microdosimetric distributions in target cells and cell nuclei: (1) image analyses methods comprising the projection of computer-generated alpha particle tracks onto magnified images of lung tissue sections and simulation of their interaction with the surrounding tissue (Diel 1978; Fritsch 2007; Hofmann 1983b; Simmons and Richards 1984), and (2) calculations of specific energy distributions for a given distance between source and target site, based on the internal microdosimetry concepts developed by Roesch (1977) (Fisher 1983; Li and Zheng 1996).

Calculations of microscopic dose distributions around PuO_2_ particles in the lungs of hamsters, rats and dogs, based on the projection of computer-generated alpha particle tracks onto lung tissue sections and their interaction with the surrounding tissue were reported by Diel (1978, 1982), Diel et al. (1984, 2007) and Fritsch (2007). As opposed to the single emissions of radon progeny alpha particles, PuO_2_ particulates are characterized by multiple emissions of alpha particles, emitted in a radial fashion. The results of such calculations were local dose rates and fractions of alpha particles which penetrate or pass through tissue as a function of the distance from a PuO_2_ particle (Diel 1978, 1982; Diel et al. 1984, 2007) or distributions of alpha particle hits per target cell (Fritsch 2007). Although these local dose distributions do not represent microscopic energy distributions in a strict physical sense, they are often referred to in the literature as microdosimetry (Diel 1982).

While the approach taken by Simmons and Richards (1981) was similar to the calculations of Diel (1978), they also provided specific energy distributions in cells and cell nuclide for a range of PuO_2_ activities in human, rat and beagle dog lungs (Simmons 1992; Simmons and Richards 1981, 1982, 1984, 1989, 1997, 2010). Thin sections of alveolar tissue were examined in an image analyzer and imaginary PuO_2_ particles were placed in an alveolar sac and radially emitted hypothetical alpha particles were tracked through tissue. In addition to plots of the specific energy as a function of the distance from the PuO_2_ particle, specific energy distributions, expressed as the volumes or numbers of cells and cell nuclei receiving a given specific energy, were computed for a wide range of alpha particle activities. In addition to the radial emission pattern around a PuO_2_ point source, Simmons and Richards (2010) also simulated specific energy distributions for randomly distributed particle tracks for the same number of accessible cells.

Similar image analysis methods were employed by Hofmann (1981, 1983b) and Hofmann et al. (1990b) to simulate the interaction of computer-generated alpha particle tracks emitted from PuO_2_ nuclides with alveolar cell nuclei on randomly selected rat lung tissue sections. Specific energy distributions were then calculated by applying image analysis techniques. The comparison between uniform and hot particle distributions of the same activity revealed that the uniform specific energy distributions are dominated by single energy deposition events, while high specific energies observed for the particulate source point to the action of multiple deposition events. These specific energy distributions were then used to calculate spatial distributions of resulting cellular radiation effects, such as cell killing and transformation (Hofmann et al. 1990b).

While the above microdosimetric approaches represent a combination of experimental and computational image analysis methods, the studies by Fisher and Roesch (1981), Fisher (1983, 1988) and Li and Zheng (1996) for PuO_2_ alpha particles are based on the microdosimetric concepts for internal radionuclide distributions developed by Roesch (1977) (see “Principles of internal microdosimetry”). The computational methods are the same as those used for the above discussed microdosimetric calculations for radon progeny alpha particles in bronchial cells, except that they refer to localized particulate sources. To consider the structure of the alveolar tissue, Fisher and Hadley (1984) developed a statistical model of the alveolar microstructure based on the analysis of tissue sections.

**Fig. 6:**
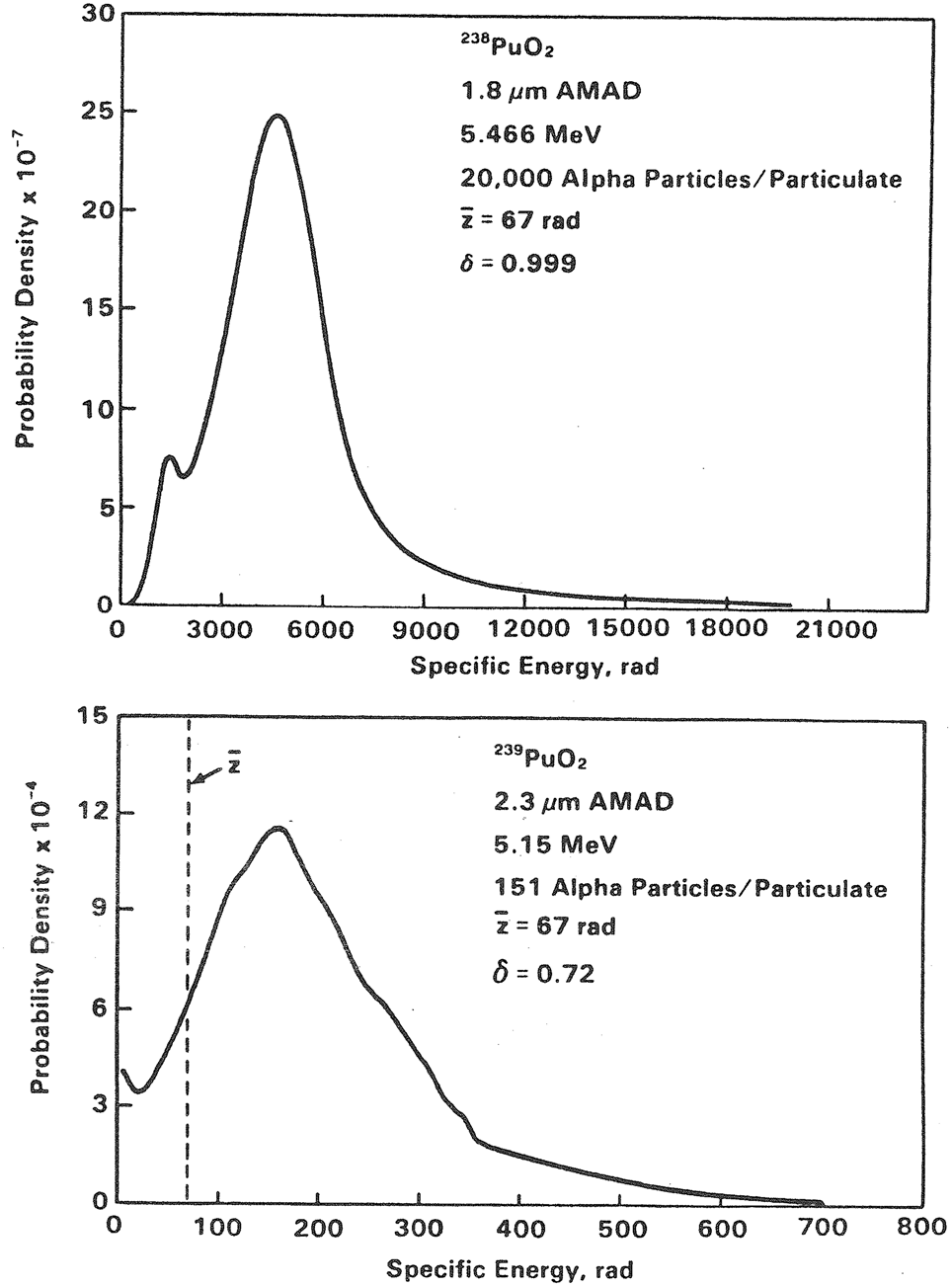
Probability density in specific energy for the beagle dog lung for the hypothetical case where one dog receives a cumulative absorbed lung dose of 0.67 Gy (67 rad) from ^238^PuO_2_ (upper panel), and another dog receives an equal lung dose from ^239^PuO_2_. Note that the mean activity of a ^238^PuO_2_ particulate is 132 times higher than the mean activity of a ^239^PuO_2_ particulate to produce the same absorbed dose. Reprinted from Fisher (1988) with permission of Oxford University Press

As an example of these calculations, specific energy distributions for the beagle dog lung from ^238^PuO_2_ (upper panel) and ^239^PuO_2_ (lower panel) are shown in Fig. 6 for the hypothetical case where one dog receives a cumulative absorbed lung dose of 0.67 Gy (67 rad) from ^238^PuO_2_, and another dog receives an equal lung dose from ^239^PuO_2_ (Fisher 1988). Due to their different half-lives, 87 vs. 24,000 years, though similar alpha energies, the mean activity of a ^238^PuO_2_ particulate is 132 times higher than the mean activity of a ^239^PuO_2_ particulate to produce the same absorbed dose. The significant differences in probability density can be attributed to differences in the respective nuclide distributions, i.e., the ^239^PuO_2_ activity is much more uniformly distributed than that from ^238^PuO_2_, which is more concentrated in “hot particles”.

## Alpha particle microdosimetry in bones

Compared to the microdosimetric calculations for alpha-emitting radionuclides in the lungs, only a very limited number of studies have been published for bone-seeking alpha-emitters. In these studies, the term localized dosimetry is sometimes used instead of microdosimetry, e.g., Polig (1978). Moreover, microdosimetry is also used as a synonym for the microdistribution of alpha-emitters in bones, e.g., Austin et al. (1999). The alpha-emitters most frequently studied are the surface seekers ^239^Pu and ^241^Am, although ^226^Ra, ^233^U, ^237^Np, and ^238^Pu were also investigated. Similar to the irradiation geometry for radon progeny alpha particles in bronchial airways, a geometric relationship exists between the activity distribution and the target cell distribution in bones. Thus, the microdistribution of the alpha-emitting radionuclides in the bone is a major determinant of the microdosimetry in bone target cells. Experimental methods to determine such microdistributions are photographic emulsion autoradiography (Polig 1978), solid-state nuclear track detector (SSNTD) autoradiography (Polig et al. 1984b; Austin et al. 1999, 2000), and, most recently, digital autoradiography using an ionizing-radiation quantum imaging detector (iQID) camera (Miller et al. 2014; Tabatadze et al. 2016) for alpha particles, and neutron-induced autoradiography (NIAR) in the case of the fissionable nuclides ^239^Pu and ^241^Am (Polig 1978; Polig et al. 1998; Austin et al. 1999, 2000).

The skeletal toxicity of alpha-emitting radionuclides is intimately related to their local distribution on a microscopic scale. A summary of microdistribution studies for ^226^Ra, ^241^Am and ^239^Pu prior to about 1975 can be found in Polig (1978). Since then, animal studies on injected ^241^Am and ^239^Pu in beagle dogs (Polig et al. 1984b, 1998) and injected ^241^Am, ^239^Pu and ^233^U in mice (Austin et al. 1999, 2000) have been reported, while the USTUR case 246 (Priest et al. 1995) refers to the ^241^Am contamination of a radiation worker in an americium recovery factory who was involved in a glove-box explosion. Due to the process of bone remodeling, the initial microdistribution of ^239^Pu, ^241^Am and ^233^U changes with time after injection (Austin et al. 1999, 2000). For example, the initial non-uniform ^239^Pu microdistribution tends to become more uniform and the activity in the spongy trabecular bone is gradually translocated to the compact cortical bone (Polig et al. 1998).

Cells lining the internal surface of mineralized tissue are considered to be the primary target cells for radiation-induced cancer in the skeleton. They are flat and their cell nuclei can be approximated by oblate ellipsoids with a diameter of 11.1 µm and a thickness of 1.6 µm (Polig et al. 1984a). The probability of the traversal of an alpha particle through the cell nucleus depends on the residence time of the cells, the activity of the alpha particle source and the spatial arrangement of both activity and target distributions.

The methodology for the calculation of alpha particle hits in bone lining cells from alpha-emitting radionuclides has first been described by Polig (1981) for spherical targets and planar alpha particle sources and later extended to oblong spheroids and cylindrical sources by Polig et al. (1992), Kruglikov and Polig (1995), and Kruglikov et al. (1993). A comprehensive description of the calculation of hit rates and radiation doses to cell nuclei of bone lining cells for alpha-emitters in humans and beagle dogs has been provided by Polig et al. (1992) for ^237^Np, ^226^Ra, ^239^Pu and ^241^Am, which are the alpha particle emitters of major interest with regard to long-term deposition and late toxic effects in the skeleton. Hit factors, which relate the local activity concentration of an alpha particle emitting radionuclide to the mean hit rate in a specified target, and related parameters, such as the dose per hit, the dose mean specific energy per hit, the mean track segment length and the percentage of stoppers were calculated using Monte Carlo methods. The irradiation geometry considers the irradiation of cell nuclei from cylindrical sources, such as the Haversian canals, and from planar sources, such as the essentially plane trabecular bone surfaces, for both surface sources and volume sources.

To illustrate the dependence of hit factors in bone lining cell nuclei on the source geometry, i.e., plane vs. cylindrical and surface vs. volume source, computed hit factors for ^237^Np, ^239^Pu and ^241^Am are compiled in Table 1. Hit factors for cylindrical sources are consistently higher than those for volume sources and they both increase with alpha particle energy. The surface/volume ratio of bone determines the comparison of relative hit frequencies from surface and volume sources of a given alpha-emitter. In the case of a volume equivalent spherical target, hit factors are correspondingly smaller than for oblate spheroids, as the flattened shape of the oblate spheroid presents a larger cross-section to alpha particle traversals from adjacent bone surfaces (Polig et al. 1992). In the case of a ^226^Ra volume source, hit factors are composed of the weighted contributions of the hit factors for the alpha-emitting decay products ^222^Rn, ^218^Po and ^214^Po. Due to the formation of new bone that buries surface deposits of an alpha-emitter, a planar distribution may be translocated into bone volume and its radiation is then partly or even fully shielded. Due to this shielding effect, hit factors decrease with increasing burial depth (Polig et al. 1992).

**Table 1:** Hit factors for ^237^Np, ^239^Pu and ^241^Am alpha particles in bone lining cell nuclei Adapted from Polig et al. (1992)

Source geometry	Radionuclide		
	^237^Np/^226^Ra	^239^Pu	^241^Am
Hit factor (10^−2^ cm^2^ Bq^−1^ day^−1^)			
Surface source			
Plane surface	4.00	4.12	4.13
Cylinder (30 µm diameter)	5.38	5.58	5.78
Hit factor (10^−5^ cm^3^ Bq^−1^ day^−1^)			
Volume source			
Plane surface	3.36	3.85	4.26
Cylinder (30 µm diameter)	4.67	5.40	6.32

Kruglikov and Polig (1995) related the number of alpha particle traversals through the nuclei of bone lining cells in trabecular bone to the chronic ingestion or inhalation of 1 ALI/year during a period of 50 years, resulting in skeletal burdens of ^241^Am, ^237^Np, ^238^Pu and ^239^Pu between 188 and 976 Bq. This continuous intake for 50 years corresponds to average hit values of 0.083 for ^239^Pu and 0.43 for ^237^Np deposits. Hit probabilities depended primarily on the type of remodeling (deterministic vs. random) and to a lesser degree on the spatial distribution of the nuclides (uniform vs. non-uniform).

**Fig. 7:**
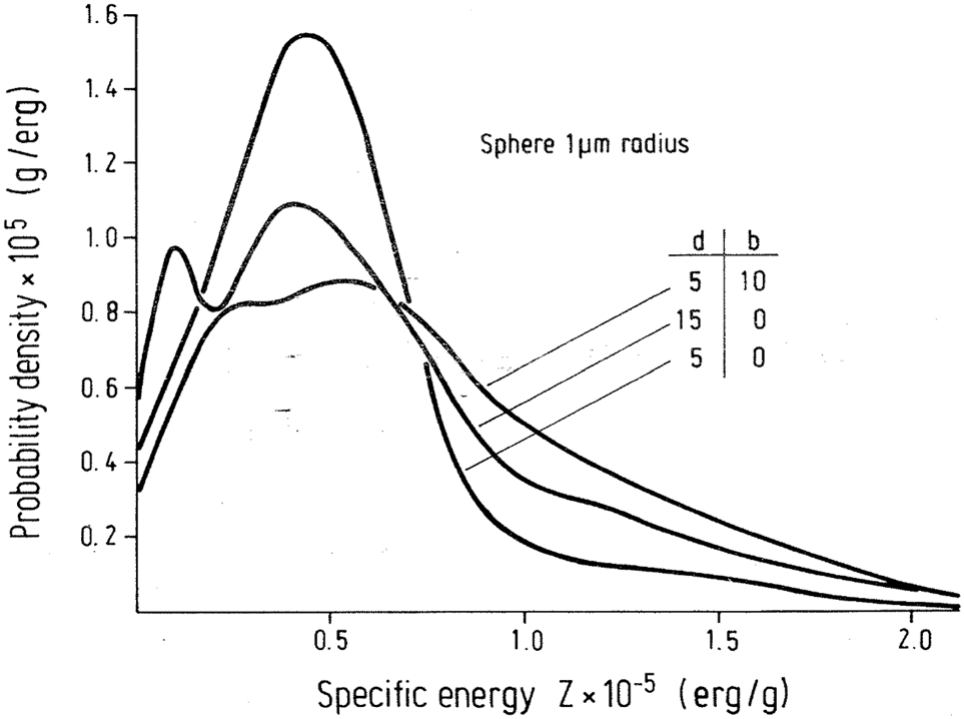
Spectra of specific energy for spherical targets from ^239^Pu alpha particles. The center of the sphere is placed at a distance *d* (µm) from the surface and the plane source of radioactivity is displaced by *b* (µm) into the bone volume (erg g^−1^ = 10^−4^ Gy). Reprinted from Polig (1983a) with permission of Springer Nature

Spectra of specific energy, *f*(*z*), in spherical and disc-shaped targets for planar sources of alpha particles were first calculated by Polig (1983a, b) applying Monte Carlo methods, considering the variation of track lengths and energy straggling for direct traversals and the contribution from δ-rays generated outside the target. For the calculation of specific energy spectra for ^239^Pu alpha particles, the theory of the microdosimetry of internal sources of Roesch (1977) was adapted to the situation of a plane alpha particle source located in bone at distance *b* from the plane bone marrow interface and irradiating a sphere or a disc at distance *d* in the marrow. Specific energy spectra for different distances of the center of a spherical target of 1 µm diameter from the surface and of the depth of the plane source from the surface of the bone are plotted in Fig. 7 for ^239^Pu alpha particles. The specific energy spectrum changes appreciably with increasing distance from the source. In addition, the variation in *z* becomes larger and the mean $\bar{z}_{1}$ increases due to the increasing LET. Figure 7 also illustrates the effect of burial, i.e., the displacement of a surface deposit into bone, by shifting the distribution to larger *z* values with increasing burial depth.

**Fig. 8:**
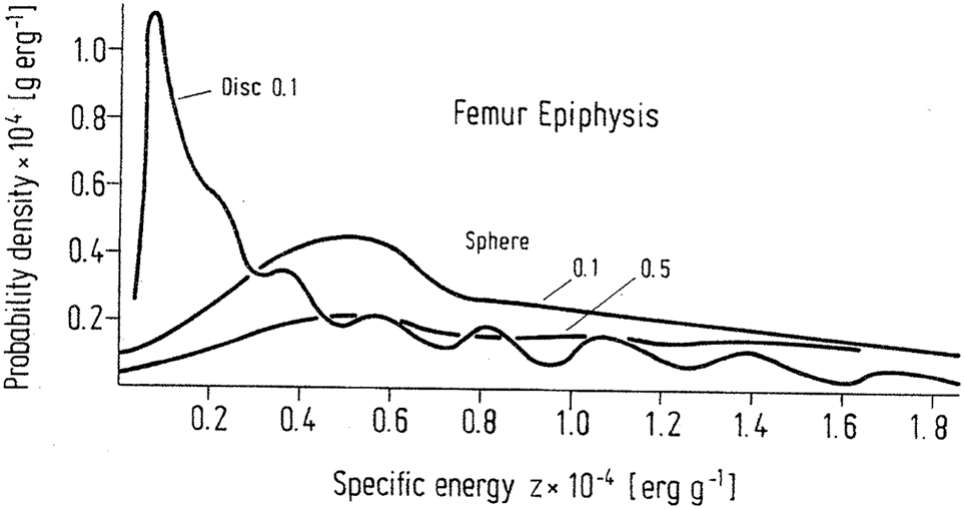
Spectra of specific energy for 13 × 1 µm discs and spheres of radius 3.16 µm touching bone surfaces that remodel probabilistically with a turnover rate of 100% year. 3.7 kBq kg^−1^ (0.1 µCi kg^−1^) and 18.5 kBq kg^−1^ (0.5 µCi kg^−1^) ^239^Pu injected intravenously in rats. Reprinted from Polig (1983b) with permission of Springer Nature

The effect of variation of surface activities as observed in autoradiographs of bone tissue sections on specific energy distributions was investigated by Polig (1983a) for ^239^Pu alpha particles emitted from a plane alpha particle source of varying intensity following ^239^Pu injection in rats. Since the irradiation time interval for these cells is determined by the remodeling activity of the skeleton, a theory of trabecular bone remodeling was incorporated into the calculation of specific energy spectra. The spectra of specific energy deposition in spherical targets with radius of 3.16 µm and disc-like targets with a radius of 6.5 and a thickness of 1 µm in the femoral epiphysis are displayed in Fig. 8 (Polig 1983a) for a turnover rate of 100% per year. The *f*(*z*) spectra for both targets reveal an appreciable influence of the geometric shape of the target, i.e., the disc has a much higher probability for low *z* values. Increasing the amount of injected activity fivefold (from 0.1 to 0.5 µCi kg^−1^) clearly lowers the probability of small *z* but only by a factor of 2. In summary, the variation of the specific energy distribution with high-level skeletal burdens is determined by the variation of the specific surface activities and the residual lifetimes as a result of bone remodeling, while it is essentially determined by Poisson fluctuations and variations of the single-event spectrum in the case of low level burdens.

Calculations of lineal energy or *y*-spectra of 5.2 MeV alpha particles in spherical volumes of different diameters are presented by Kappos (1968). Alpha particles were emitted from a contamination of a surface layer of bone, while the spherical volumes were located in adjacent soft tissue at varying distances from the layer; such an exposure situation is characteristic of surface-seeking isotopes like ^239^Pu and ^241^Am. In the intermediate vicinity of the surface, the most probable event is close to the mean LET and the mean track length through a sphere (136 keV µm^−1^). The distributions then flatten out for larger distances, adopting approximately constant probabilities over the whole y-spectrum. At large distances near the end of the alpha particle range, a peak appears at small lineal energies y due to incomplete passages (stoppers).

## Alpha particle microdosimetry in targeted radionuclide therapy

### Targeted radionuclide therapy

In targeted radionuclide therapy (TRT), alpha- beta- and Auger-emitters, which are conjugated with or without targeting molecules, are directed against tumor cells. In this context, dosimetry can be implemented: (1) to study and understand the response radiobiology at the cellular level; (2) to test several therapeutic options and to evaluate their impact of efficacy/toxicity for preclinical targeted radionuclide therapy; (3) to modulate the administered activity, to achieve patient-specific treatment in personalized medicine. To assess this kind of radionuclide therapy and the controlling potential toxicity to the patients, the cellular absorbed doses to tumorous cells and healthy neighboring cells are needed (Williams et al. 2008; Jadvar 2017; Zukotynski et al. 2016; Allen et al. 2014).

These targeted radionuclides emit short-range radiation and deliver therapeutic radiation to individual tumor cells while maintaining the irradiation to the surrounding normal tissues below the threshold of toxicity. However, the dosimetry of these radionuclides is challenging, because the absorbed dose may have to be characterized on a scale that is comparable to the range of these emissions, i.e., millimeters for beta particles, micrometers for alpha particles, and nanometers for Auger electrons (Roeske et al. 2008). Therefore, it needs to implement and quantify the dosimetry at a small or even microscopic scale. Microdosimetry takes into account the stochastic nature of energy deposited in small targets for alpha particle dosimetry. The necessity for microdosimetric methods depends on the source distribution, the target size and shape, and the expected mean absorbed dose. For example, a small cell nucleus with a diameter of 5 μm irradiated by alpha particles would require an average absorbed dose of at least 100 Gy for the relative deviation to be less than the 20% threshold, at which the fluctuations around the mean are so important that the average absorbed dose is no longer the parameter that determines the observed biological effect (Sgouros et al. 2010).

The microdosimetric approach proposed by Stinchcomb and Roeske (1992) led first to the determination of *f*_1_(*z*), the single-hit distribution in the nucleus that gives the probability of having a specific energy *z* (in Gy) imparted to the nucleus, for one and only one particle hit in the nucleus. Interestingly, *f*_1_(*z*) is specific to the given geometry and the type of radiation. In that sense, it could be considered as the microdosimetric analog of the MIRD S value (Loevinger et al. 1991).

The calculation of the specific energy spectrum *f*(*z*;*D*) can be calculated by compound Poisson process (Eq. 9) by convolution of *f*_1_(*z*) and taking into account the probability of particle hits, it also allows to determine the average number of hits, *M*. As already shown in Fig. 3, the specific energy spectrum changes with dose, i.e., with the number of emitted particles. For example, in the work of Humm (1987), Stinchcomb and Roeske (1992) and Bardiès (2011), if ^211^At were labeled on the cell surface, approximately 1/15 alpha particle out of 25 alpha particles emitted actually hits the nucleus and thus the average particle hit, *M* is 1.67. This means that the most prominent peak in the spectrum represents the situation of one hit in the nucleus, and to a lesser extent the peak of two hits in the nucleus. Increasing the number of emitted particles has two consequences: the first is to increase the average specific energy; the second is to change the shape of the specific energy spectrum, exhibiting distinct peaks for one, two and more hits. Since the relative deviation around the average specific energy is going to decrease for a certain number of particles emitted, the conditions to perform a macrodosimetric analysis (relative deviation < 20%) will be met. Thus, it will be possible to use the absorbed dose, i.e., a non-stochastic parameter, to describe the energy deposition pattern within the irradiated material (Bardiès and Pihet 2000).

**Fig. 9:**
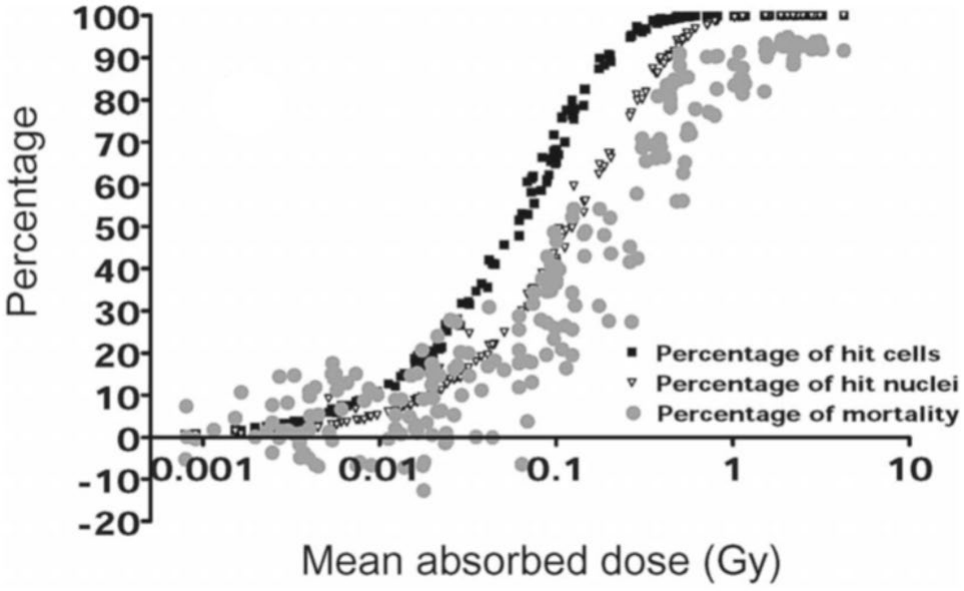
Percentage of mortality and percentage of hit nuclei or whole cells vs. absorbed dose to the whole cell (Chouin et al. 2009b). Adapted from Chouin et al. (2009b) with permission of the Radiation Research Society

In addition, the use of macrodosimetry and microdosimetry may be dictated by the experimental context and the biological outcome. In some situations, the microdosimetric approach should be warranted and macrodosimetric results may be sufficient for the intended purpose (ICRU 1983). For example, in the work of Chouin et al. (2009a, b), a microdosimetric model was developed to account for the survival of lymphoid cell lines irradiated with ^213^Bi-labeled monoclonal antibodies. Single hit-specific energy spectra were developed for the geometry of the two cell lines (Ada and T2) considered. However, it turned out that the representation of the survival fraction as a function of the absorbed dose (non-stochastic parameter) was sufficient to answer the question of the identification of the relevant target volume (whole cell or cell nucleus). Furthermore, plotting survival fraction and number of hit cells/nuclei on the same graph allowed identifying domains where some mortality was observed independently of the number of hits (i.e., where non-irradiated cells were actually dying), thereby hinting at a bystander effect (see Fig. 9).

For small absorbed doses, such as those expected by non-targeted tissues, microdosimetry may be important in characterizing the pattern of energy deposition and in understanding how this pattern is related to clinical outcomes (Sgouros et al. 2010).

### Small-scale and microdosimetry in targeted radionuclide therapy

**Fig. 10:**
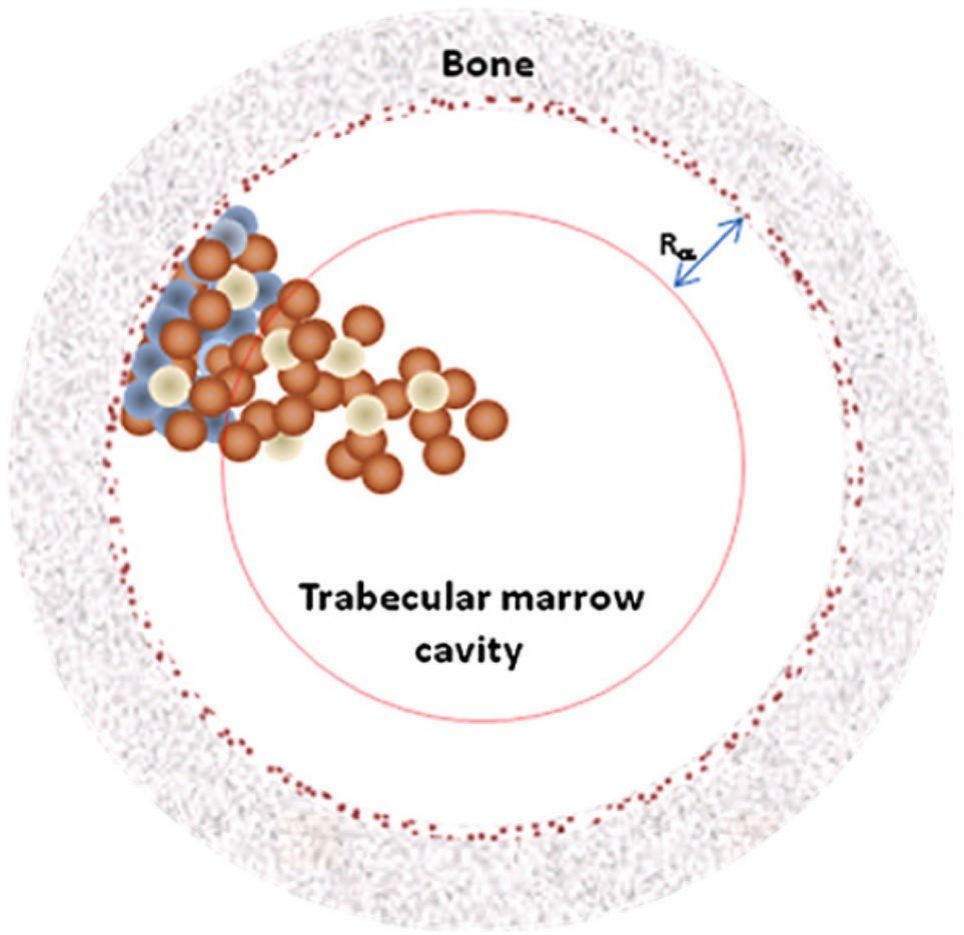
Representation of the marrow cavity model. The cavity is represented by a sphere of radius *R*_c_. *R*_α_ is the range of the α-particles from ^223^Ra decay. The blue spheres are osteoprogenitor cells, while the brown spheres are hematopoietic stem and progenitor cells and the white spheres are adipose cells. The 10 μm endosteal layer is represented by the brown speckled ring. Reprinted from Hobbs et al. (2012) with permission of IOP Publishing (color figure online)

Microdosimetry has been applied in the radionuclide therapy, especially in radioimmunotherapy (Humm 1986, 1987; Humm et al. 1993). Akabani et al. (2003) carried out microdosimetric analyses for the treatment of EMT-6 lung tumor colonies in nude mice with lung histological images and autoradiography data for microdistribution and alpha particle Monte Carlo transport and evaluated survival fraction-based microdosimetric distributions. Hobbs et al. (2012) recently simulated cellular-scale dosimetry by creating simple spheres representing marrow cavities and positioning ^223^Ra on the trabecular bone surface or in the endosteal layer (Fig. 10). The interior of the sphere was divided into cell-size voxels and the energy was collected in each voxel and interpreted as absorbed dose cell histograms. The results from the marrow cavity model differ markedly from a standard absorbed fraction method which represents average absorbed dose values. The marrow cavity model offers an explanation for the clinical evidence suggesting that the average absorbed dose will not reflect biological outcome in the case of ^223^Ra therapy.

Amato et al. (2015) developed a computational model of solid tumor microenvironment around a blood capillary vessel, and simulated the transport of radiation emitted by ^223^Ra, ^111^In, ^131^I and ^177^Lu using the Geant4 Monte Carlo code. For each nuclide, several models of radiopharmaceutical dispersion throughout the capillary vessel were considered. This microdosimetric approach can quantify absorbed dose distributions at the microscopic level around a simple model of a tumor capillary vessel for some therapeutic radionuclides by taking into account the differences between irradiation properties of alpha, beta and Auger emissions. The results can help to characterize the absorbed dose inhomogeneities in solid tumor therapies with radiopharmaceuticals, taking into account the interplay between drug distribution from vasculature and range of ionizing radiations.

Although not directly related to alpha-emitting radionuclide therapy, microdosimetry is continuing to play an important role in the radiotherapy for determining the RBE for high-LET radiations and for low-LET radiations as well (Wambersie et al. 1990). The recent application of gold nanoparticles in radiotherapy by X-rays and proton beams raise the question of RBE values for this new preclinical radiation therapy. A study showed that the effectiveness of proton radiotherapy for the killing of prostate tumor cells was increased by approximately 15–20% for those cells containing internalized gold (Polf et al. 2011). Microdosimetry is needed for redefining the RBE for emerging alpha particle radiopharmaceuticals (Hobbs et al. 2014; Kratochwil et al. 2017).

### Quantitative digital autoradiography

As the number of proposed targeted radionuclide therapies using alpha-emitters expanded (Sgouros et al. 2010; Sgouros 2019), tools are needed for preclinical and clinical studies that provide spatial dosimetric information near cellular levels to optimize the therapeutic response to targeted tissues and accurately estimate the absorbed doses to normal organs. The exact determination of the microdistribution of alpha-emitting radionuclides is a necessary condition for a successful radiotherapeutic application of alpha-emitters. For example, quantitative digital autoradiography has been used for the measurement of the ^241^Am microdistribution in bones (Tabatadze et al. 2016) (see “Alpha particle microdosimetry in bones”).

The current set of imaging tools that are available to researchers including scintigraphy, film autoradiography, and SPECT/CT do not allow for an effective assessment of radiation absorbed dose distributions at cellular levels, because resolutions are poor, measurement and analytical times are long, and the spatial resolutions are low—generally resulting in poor signal-to-noise ratios. In the case of autoradiography with phosphor imaging plates, activity quantification requires the use of standards, which may have high uncertainty at low activity levels, and lacks any real-time imaging capability that can be used to assess whether or not sufficient signal has been acquired for the image study. Additionally, since these systems integrate scintillation light from all events, the final image has an inherent blur due to the light spread from each event interaction (Miller et al. 2015).

Recently, new digital imaging tools have been developed that provide ex vivo assessment of the spatial radionuclide concentrations at resolutions approaching cellular levels (~ 20 μm) and quantification at millibecquerel per microgram levels. A review of these single-particle quantitative digital autoradiography technologies for both alpha and beta emitters has recently been published (Miller 2018). A distinguishing feature of these imaging detectors is their high detection efficiency (~ 100% for alpha particles) and ability to estimate the emission location of individual particles on an event-by-event basis and construct the radioactivity spatial distribution in real time.

Another detector, that has been integral in multiple preclinical targeted radionuclide therapy studies is the alpha-camera (Bäck and Jacobsson 2010; Sgouros et al. 2010). Although not a single-particle imaging detector, this digital scintillation-based imaging detector offers high spatial resolution, real-time monitoring during image acquisition, and activity quantification when combined with a gamma-ray counter.

**Fig. 11:**
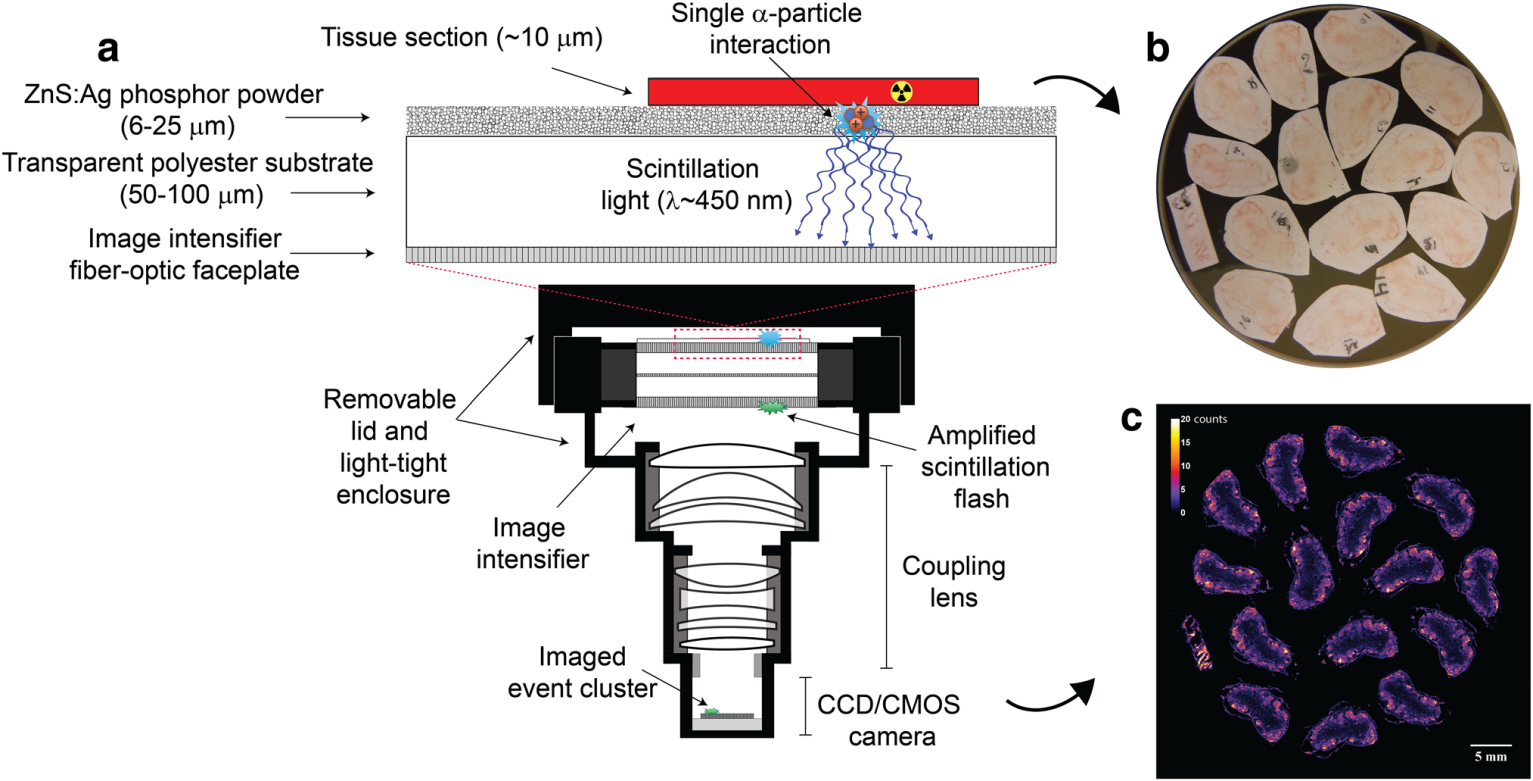
**a** iQID schematic/setup for quantitative digital autoradiography with alpha-emitters, **b** iQID optical view of biopsy tissue sections placed on a 40 mm diameter iQID detector and **c** corresponding digital autoradiograph

**Fig. 12:**
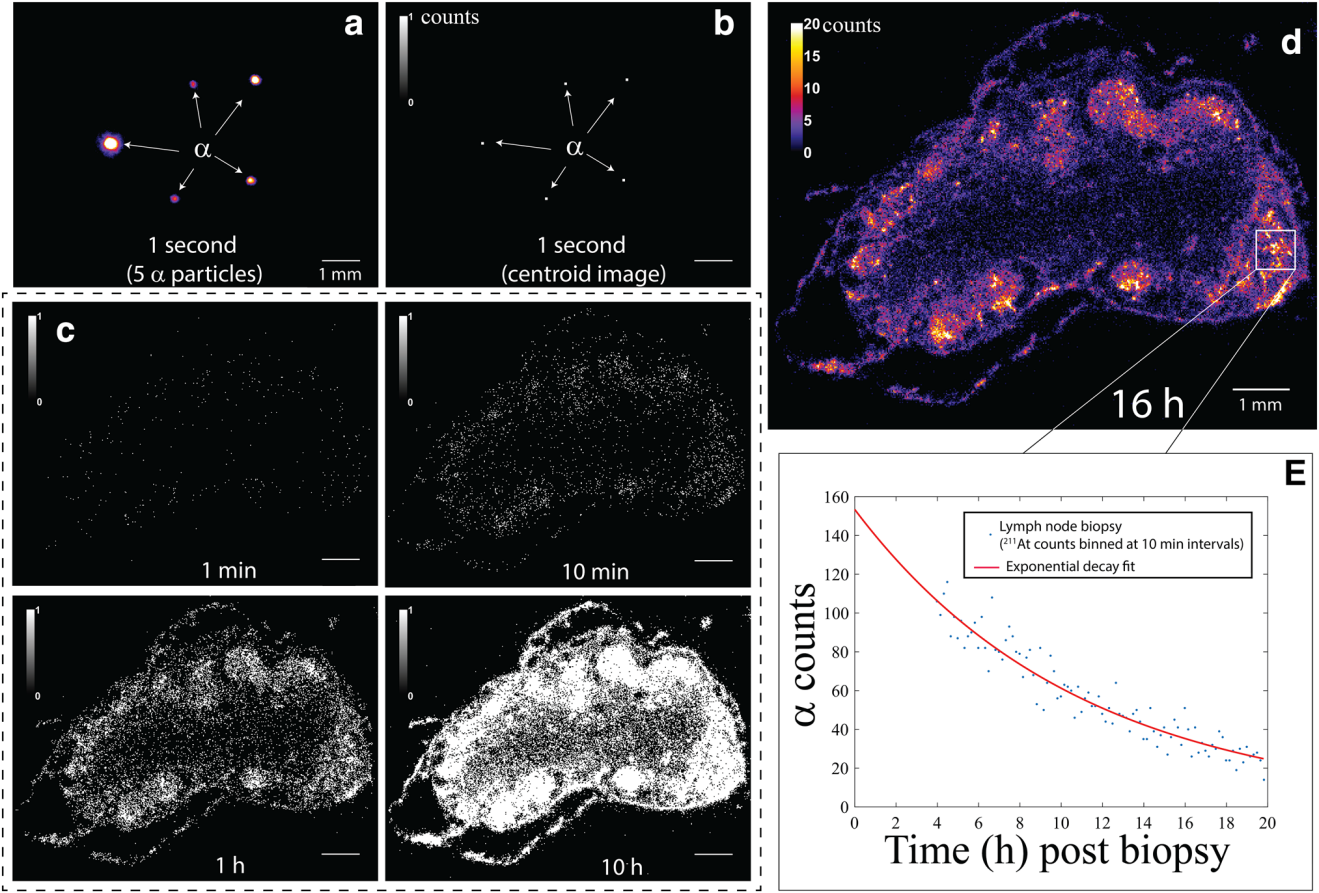
**a** One-second iQID image of ^211^At alpha particles from a biopsy tissue section and **b** corresponding centroid autoradiograph. **c** iQID biopsy autoradiographs at various time points displayed with a binary colorscale. **d** Final 16 h autoradiograph and **e** temporal information for a small region of interest, where the estimated total activity of the section ROI at the biopsy extraction time point is 477 mBq

To visualize the radionuclide distribution at high resolution, sample preparation typically involves slicing sections of excised tissue, from a biopsy or necropsy, into thin sections that are then placed on a microscope slide. For scintillation-based systems, the sample is placed in direct contact with a scintillator screen or a thin layer of mylar. For alpha particle imaging in scintillation-based systems, tissue sections can also be placed directly on a ZnS:Ag scintillation screen as illustrated in Fig. 11. As individual events are detected, an image of the radionuclide distribution is constructed in real time. This is illustrated in Fig. 12 for an iQID system, where pixel values in the image correspond to the number of alpha particles detected at a given location which can be used to estimate the activity at the biopsy or necroscopy time point for short-lived isotope like ^211^At.

## Alpha particle tracks in silico: from nanoscale to microscale

It is generally accepted that relevant radiation effects in mammals are mostly due to damage in individual cells: mutations, chromosomal aberrations, transformations and loss of proliferative capacity (cell death). In particular, genomic DNA inside the cell nucleus is supposed to be the most important target (UNSCEAR 2012). Alpha particles liberated by decay processes of radionuclides represent a radiation quality with very high effectiveness. This is reflected for radiation protection purposes in the quality factor of 20 (ICRP 2007) which may be taken as an upper limit of the relative biological effectiveness (RBE) for a variety of biological endpoints. Ionizations, and to a minor degree, excitations make up the initial condition of a radiation insult. For a given absorbed dose, the number of ionizations per cell or per cell nucleus or within the chromatin is almost the same for all types of radiation. However, their distribution on different spatial scales, the so-called track structure, is quite different and thus closely related to the biological effectiveness. The identification of parameters of physical track structure that predominantly determine the nature and magnitude of a radiation effect is one of the objectives of radiation track structure theory (Paretzke 1987). Such concepts and parameters are needed on the one hand for practical applications of radiation such as radiation therapy and on the other hand for understanding the underlying mechanisms.

When looking for the relevant scales of the biologically critical damage, it has to be taken into account that radiation tracks have a complex stochastic structure and that the genetic material in the cell nucleus has a detailed architecture, both spanning scales from 10^−10^ to 10^−5^ m. The first issue is addressed by Monte Carlo track structure codes, the second one by sophisticated models of nuclear DNA and chromatin.

Track structure calculations provide an in silico representation of the interaction patterns of energetic particles in space and time. These interactions include elastic scattering processes where only the transport direction of the particle changes, and inelastic processes where energy is transferred to the medium and to secondary liberated energetic particles, most frequently to an electron due to an ionization event. Between these interactions, the particle travels along a straight line, thus, a classical view of the nanoscale scenery is adopted. The stochastic nature of the radiation tracks comes into play via a sequence of pseudo-random numbers that determine, alternately, transport distances and characteristics of the interactions from corresponding distributions based on total and differential cross-sections, respectively. Cross-sections, the physical quantity for the magnitude of a certain type of interaction, are the core of track structure calculations; they depend mainly on the type of the ionizing particle, its energy and the traversed material. Therefore, a crucial condition for the development of event-by-event Monte Carlo track structure codes was the availability of total and differential cross-sections for interactions of the energetic particles. The differential referred to the type of interaction (excitation, outer and inner shell ionization), the energy transfer to the medium and a secondary (or even tertiary) particle (and the angular scattering of the primary and emission direction of a secondary particle.

The era of Monte Carlo track structure calculations began with the pioneering work of Berger (1963). A few years later, the needed highly differential cross-section data sets became available for water from theoretical considerations and experimental investigations. This started for electrons (Opal et al. 1972; Vroom and Palmer 1977; Paretzke and Berger 1978), which are also of prime importance for alpha particle track structures, and was advanced to protons (Miller and Green 1973; Toburen and Wilson 1977) and alpha particles (Toburen et al. 1980; Rudd et al. 1985). Correspondingly, track structure codes have been developed based on cross-section for water vapor with density scaling (Wilson and Paretzke 1980) and liquid water (Hamm et al. 1985) as a material being also representative for biological matter. In the following, further cross-section data sets have been determined including charge-changing processes for protons and alpha particles (Dingfelder et al. 2000; Uehara and Nikjoo 2002; Uehara et al. 2001; Friedland et al. 2005); detailed information about cross-sections for track structure calculations is available elsewhere (Nikjoo et al. 2012, 2016). In parallel, several track structure codes were developed for simulating biological radiation effects (Nikjoo et al. 2006) as counterpart of a number of radiation transport codes that assess the modification of the radiation during transport in matter.

**Fig. 13:**
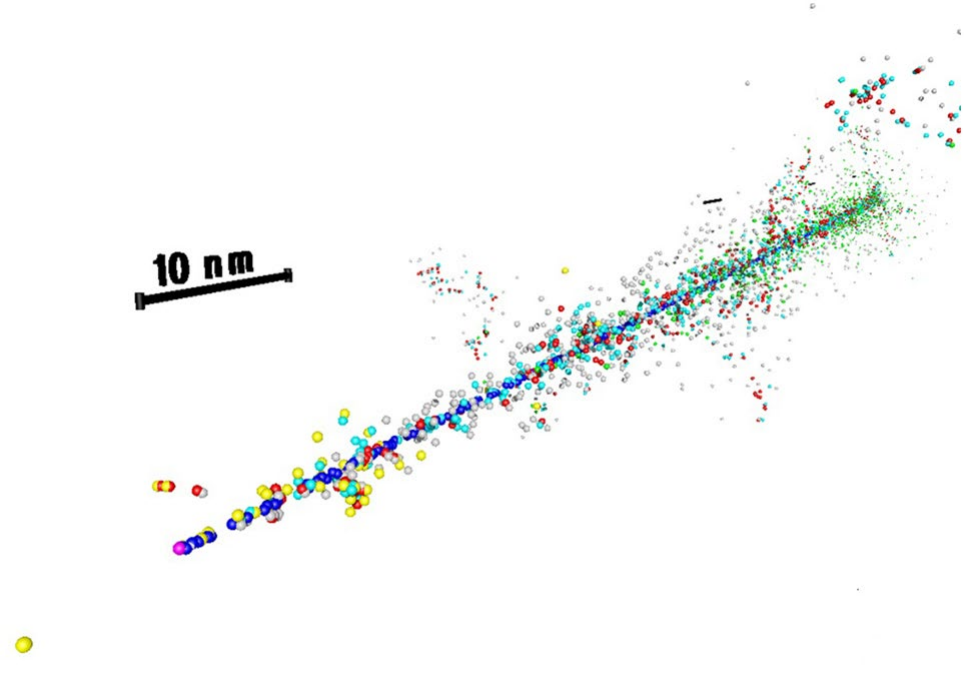
Perspective view of an alpha particle track simulated by PARTRAC. The primary particle has entered the scenery with 4 MeV energy at right end and moved about 0.4 µm during 30 fs towards the foreground on the left. Energy deposition events and moving electrons are represented by small spheres with diameters inversely proportional to the distance from the viewpoint. Ionizations and excitations by the primary alpha particle (blue) and charge-changing processes (pink) are surrounded by excitations (cyan) and some ionizations (red) due to secondary electrons. At larger distances, electrons with energies below 10 eV (gray) are slowing down to hydrated electrons (green). Electrons moving with more than 10 eV energy (yellow) are rare at these low primary particle energies. The 10-nm scale bar is repeated along the track each 100 nm up to 500 nm from the leftmost one. Visualization by PoV-Ray™ ray tracer software (Persistence of Vision Raytracer Pty. Ltd., Williamstown, Victoria, Australia) (color figure online)

Two-dimensional projections of electron and ion tracks provide an instructive view at the nanoscale distribution of energy deposition in its dependence on electron energy, ion energy and ion type (Paretzke 1987). Ray tracer software calculations on the energy deposition scenery offer improved illustrations of the track structures (Friedland and Kundrát 2014). Figure 13 shows a perspective view of a α-particle during slowing down from 4 MeV. Moving the viewpoint in subsequent pictures by the same speed as the primary particle gives a three-dimensional impression of the dynamics of track formation.

First applications of track structure calculations in radiation biology were aimed at retrieving the amount of energy deposition or the number of ionizations needed within a certain nanoscale target volume for some biological endpoint, such studies began in the 1950s (Hill 2018). In such an investigation (Goodhead and Charlton 1985) clusters from ion irradiation with at least 340 eV energy deposition within a cylindrical volume with 10 nm diameter and 5 nm height correlated with the LET-dependent frequency of lethal lesions. Corresponding distributions of energy depositions in nanometric cylinders were comprehensively determined for a variety of radiation qualities by Nikjoo and Liamsuwan (2014).

**Fig. 14:**
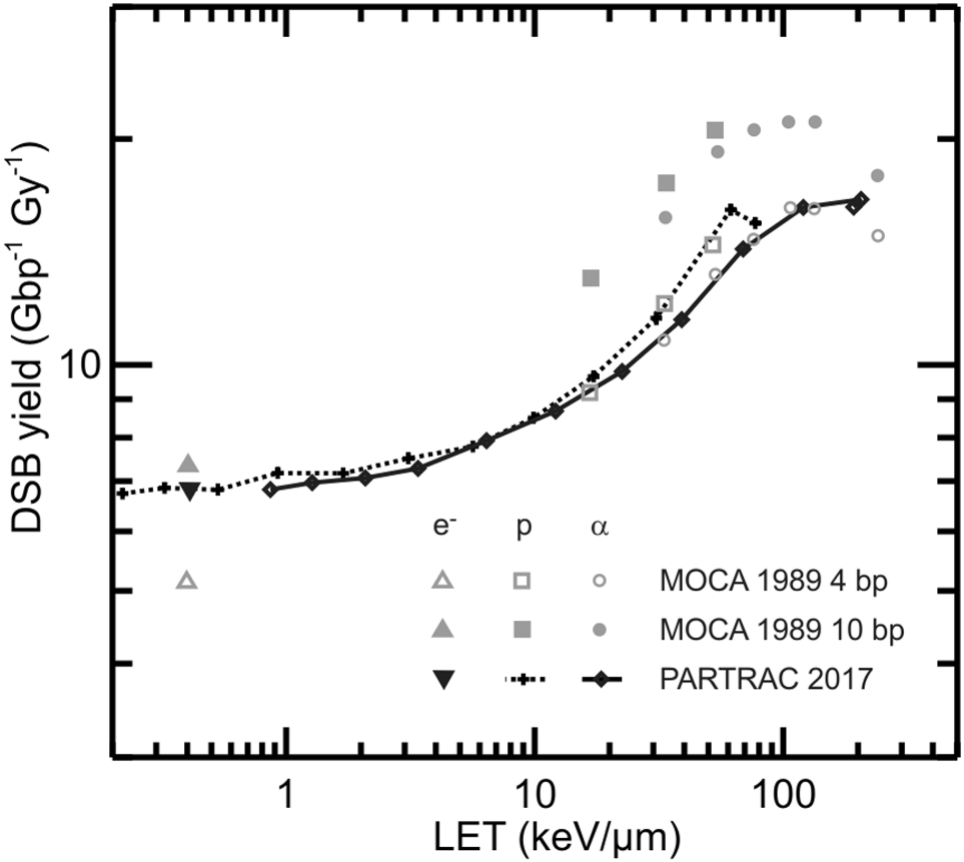
Calculated yields of DSB after low-LET electron, proton and alpha particle irradiation. Gray symbols: MOCA calculation (Charlton et al. 1989) with 4 bp (open symbols) and 10 bp (closed symbols) maximum distance of breaks on opposite strand. Black symbols and lines: PARTRAC calculation (Friedland et al. 2017) with 10 bp maximum distance of breaks on opposite strands; multiple DSB within 25 bp distance are not resolved and scored as isolated ones. Result for electrons taken from calculations for ^60^Co gamma rays (Friedland et al. 2019)

Genomic DNA has a double-helix structure with the two complementary sugar-phosphate strands being about 2 nm apart from each other surrounding the nucleobases in between. This structure has first been considered by subdividing the DNA cylinder of 2.3 nm diameter into a central cylinder of 1 nm diameter and two arches rotating by 36° after 0.34 nm height corresponding to a nucleotide pair (Charlton and Humm 1988). This simple DNA helix model was used to determine single-strand break (SSB) and double-strand break (DSB) induction after electron (20, 1.5 and 0.28 keV), proton (2, 0.75, 0.38 MeV) and alpha particle (10, 6, 4, 3, 1.2 MeV) irradiation (Charlton et al. 1989) based on energy depositions from MOCA track structure codes (Wilson and Paretzke 1981; Paretzke 1987). Essential results regarding alpha particles were: (1) decreasing SSB yield with increasing LET; (2) maximum DSB yield of 3.2 × 10^−11^ DSB per Gy and Dalton around 100–120 keV µm^−1^ LET (3–4 MeV) about 3 times the value for 20 keV electron low-LET radiation; (3) at the same LET slightly lower DSB yields for alphas than for protons. These results were confirmed in later studies (Nikjoo et al. 1999; Friedland et al. 2005), and a recent analysis showed the same maximum DSB yield for alpha particles (Fig. 14), however, at the maximum possible LET of about 220 keV µm^−1^ (Friedland et al. 2017).

This DNA model (Charlton and Humm 1988) has been adopted in several further studies on radiation damage to DNA (Ottolenghi et al. 1995; Nikjoo et al. 1999). In parallel, atomic models of DNA started to be developed and used starting from a linear piece of DNA (Pomplun 1991) and later extended to nucleosomes (Pomplun and Terrissol 1994), 30-nm fiber structures (Holley and Chatterjee 1996), chromatin fiber loops (Friedland et al. 1998) and chromosomes (Friedland et al. 1999) as higher-order DNA structures. Nowadays, a sophisticated description of many structural DNA levels inside the cell nucleus is available for many track structure codes (Friedland et al. 2008; Nikjoo and Girard 2012; Meylan et al. 2016; Li et al. 2015).

The consideration of higher-order DNA structures was essential for non-random DNA fragmentation (Rydberg et al. 1998; Friedland et al. 1999; Bernhardt et al. 2003). Moreover, it is important for the response of the cell to DNA damage, in particular the formation of chromosome aberrations where both the genomic location of the DSBs on the chromosomes and their proximity within the cell are important factors. Investigations on the mechanisms underlying chromosomal aberrations based on proton and alpha particle irradiation experiments supported the hypothesis that complex DNA lesions were critical lesions (Ballarini et al. 1999); however, their clustering on regional chromatin and large chromosome scales was not taken into account. This restriction was overcome with a DNA damage response simulation based on the qualitative and spatial distribution of initial DNA lesions taking into account the chromosomal architecture inside a human cell nucleus (Friedland et al. 2011). Application to the induction of dicentrics (Friedland and Kundrát 2013) from alpha particles revealed the slightly sublinear increase with dose—unlike the linear-quadratic dependence after photon irradiation—but without parameter adaptation the absolute dicentric yields were overestimated. The formation of chromosomal aberrations is also addressed by the BIANCA approach, which considers the cell killing effect as well (Ballarini and Carante 2016; Carante et al. 2018). The consideration of clonogenic cell survival/cell killing in parallel with chromosomal aberration induction is of particular interest for improved radionuclide therapy with alpha-emitters.

Track structures of alpha particles on nanometer scale are characterized by mean free paths of 0.1–1 nm between subsequent ionizations. The limited energies of secondary electrons with a maximum of about 4 keV confines the energy deposit close to the alpha particle trajectory: 55% are located within 1 nm, ~ 90% within 10 nm radial distance. Both these issues lead to an unexcelled effectiveness of alpha particle radiation in producing DSB sites (Friedland et al. 2017, 2018); more densely ionizing radiation by heavier ions is supposed to yield overkill effects.

Track structures of alpha particles on micrometer scale are characterized by their range between ~ 27 and ~ 95 µm in liquid water and tissue for energies between 4 and 9 MeV, respectively. Along this path, the linear energy transfer rises to its maximum (about 225 keV µm^−1^) at ~ 5 µm, but stays above ~ 55 keV µm^−1^ at up to ~ 0.6 µm before stopping; high-LET radiation effects are thus observed along the whole track. In view of typical cellular dimensions, the range leads to direct radiation effects in multiple cells; depending on the microstructure of the tissue, even multiple cell nuclei are frequently hit by a single track. The consequence of such coincident damage in neighboring cells is not known. However, even without involvement of multiple cells, the impact of alpha particles on intercellular communication became clear when low fluences of alpha particles gave rise to bystander effects (Azzam et al. 1998) resulting in considerable modification of gene expression. Thus, track structure-based investigations of biological effects due to internal alpha-emitters are an important issue on the radiation research agenda.

## Relationship between microdosimetry and cellular radiobiological effects

Results of radiobiological in vitro studies with alpha particles for different endpoints, such as cell killing (or inactivation), mutation, transformation, or micronucleus formation, are commonly presented as functions of dose, while alpha particles are characterized by their LET. Thus, at present, cellular radiobiological effects cannot be directly predicted solely on the basis of computed specific energy distributions, nor can these predictions be validated by comparison with experimental data. Despite this fundamental problem, several substitute microdosimetric approaches were proposed to establish such a relationship, such as hit-related concepts, effect-related track length models, effect-specific interpretation of specific energy distributions, and, finally, models based on track structure calculations. The biological effects most relevant for radiation-induced carcinogenesis are oncogenic transformation and cell killing (or cellular survival).

### Hit-related concepts

Target cell nuclei may receive single or multiple hits from alpha particles. Thus, the basic hypothesis of hit-related concepts is that biological effects are related to the fraction of cells hit by alpha particles, and in the case of a hit, to the number of cellular alpha particle hits. In line with this, Fritsch (2007) proposed the distribution of the number of alpha particle hits as a new parameter for the assessment of cancer risk for ^238^PuO_2_ and ^239^PuO_2_ point sources in the human respiratory tract.

**Fig. 15:**
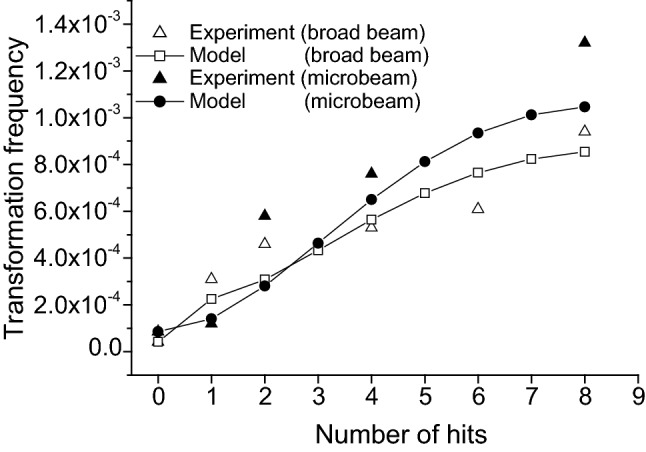
Comparison of simulated in vitro transformation frequencies per surviving cell of C3H 10T1/2 mouse embryo fibroblasts produced by a varying number of 5.3 MeV alpha particles with experimental data for microbeam and broad beam irradiation conditions. Adapted from Fakir and Hofmann (2006)

Based on the generalized state-vector model of radiation carcinogenesis (SVM) (Crawford-Brown and Hofmann 1990), which includes single-track characteristics of charged particles with varying LET, Fakir and Hofmann (2006) simulated the relationship between in vitro transformation frequencies per surviving cell of C3H 10T1/2 mouse embryo fibroblasts irradiated by charged particles with a wide range of LET values (Miller et al. 1995). For example, predictions of transformation frequencies per surviving cell produced by a varying number of 5.3 MeV alpha particles (LET = 90 keV μm^−1^) are compared in Fig. 15 with experimental data for microbeam and broad beam irradiation conditions (Miller et al. 1999), exhibiting a non-linear relationship at higher hit numbers. While microbeam irradiation represents exposure of cell nuclei to exact numbers of alpha particle traversals, broad beam irradiation represents exposure to a Poisson-distributed number of alpha particle traversals for a given average traversal number.

Balásházy et al. (2009) investigated the effect of radon progeny accumulations at airway bifurcations and the resulting distribution of single and multiple hits on transformation probabilities. Even at low average tissue doses, most cells located at carinal ridges receive multiple hits, eventually leading to a non-linear relationship between cellular hits and transformation probabilities.

### Effect-specific track length model

Since range and energy straggling as well as the contribution from *δ* rays produced by grazing alpha particles can be neglected for micrometer sites, such as cell nuclei, the specific energy distribution for single hits, *f*_1_(*z*), for a given alpha particle energy can be approximated by the track length distribution of intersecting alpha particles and the lineal energy distribution *f*(*y*) can be replaced by the LET distribution (Hofmann 1982). Furthermore, irradiation conditions in radiobiological in vitro studies are commonly characterized by the LET of the incident radiation. In the effect-specific track length model (Crawford-Brown and Hofmann 2001, 1991; Hofmann et al. 2000b), the random intersection of an alpha particle through cell nuclei and the multiplicity of cellular traversals for a given dose are related to a specific radiobiological effect probability through the so-called probabilities per unit track length (PPUTL). These probabilities per unit track length of observing a specific biological radiation effect, such as cell killing or transformation, upon traversal of an alpha particle of a given LET through a cellular target were derived from experimental in vitro experiments (Cox et al. 1977; Thacker et al. 1979; Hieber et al. 1987; Miller et al. 1995). Note that the PPUTL for transformation is correlated with the PPUTL for cell killing, as only surviving cells can express an oncogenic transformation. The effect-specific track length model has been applied to radon progeny activities on bronchial airway surfaces in the human lung (Hofmann et al. 1992, 1994, 2000b, 2002; Fakir et al. 2006; Lau et al. 2006) and to highly localized plutonium alpha particle sources in the alveolar region of the rat lung (Hofmann et al. 1990b).

The application of the effect-specific track length model for the evaluation of the carcinogenic potential of inhaled radon progeny in human bronchial airways required the following computational steps (Hofmann et al. 2000b): first, LET spectra for ^218^Po and ^214^Po alpha particles in cell basal and secretory cell nuclei located at varying depths in bronchial epithelium were computed for selected airway generations. Second, since bronchial cells at a given depth experience a wide distribution of LETs, experimentally derived transformation and cell killing PPUTLs for selected LET values were expressed by continuous functions of LET. Third, probabilities for oncogenic transformation were determined for individual alpha particle traversals by applying the effect-specific track length model. Finally, transformation and cell killing probabilities as functions of dose were calculated, based on the chord length distributions for alpha particles traversing a spherical target (crossers) or stopping in the nucleus (stoppers), as well as the number of nuclear traversals as a function of dose and the relative frequencies of basal and secretory cells. Calculations revealed that in contrast to the nearly linear decrease of tissue doses with increasing depth in bronchial tissue, transformation probabilities for basal and secretory cells exhibit distinct maxima at their preferential locations in bronchial epithelium, where the highest transformation probabilities are associated with the deeper lying basal cells.

To explore the carcinogenic response of non-uniform alpha-emitting radionuclide distributions as compared to the same activity of uniformly distributed alpha particle sources, the PPUTL concept was applied to radon progeny accumulations at bronchial airway bifurcations, using single (Szőke et al. 2006; Farkas et al. 2011) and multiple bifurcation models (Szőke et al. 2008, 2009). A slightly modified approach was adopted by Szőke et al. (2012) who combined track length distributions with experimentally derived cell killing and transformation functions based on average cellular doses to evaluate the contributions of bystander cells to the transformation probabilities in airway bifurcations as compared to direct hits.

**Fig. 16:**
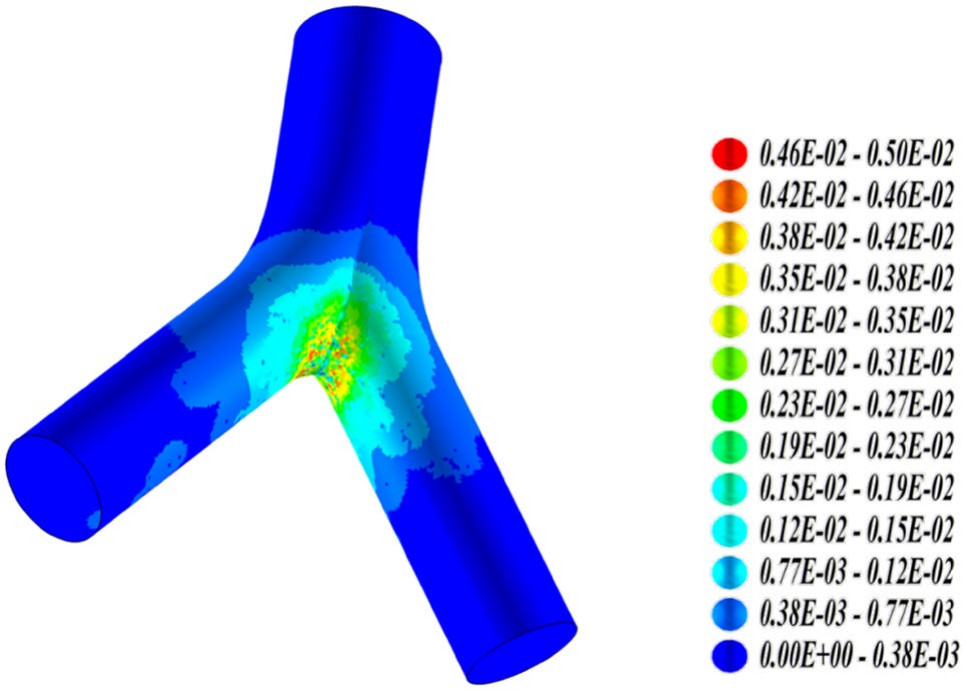
Distribution of transformed basal and secretory cells in a bronchial airway bifurcation model following exposure to radon progeny alpha particles for defined environmental and physiological inhalation conditions, based on direct hits plus the indirect contribution of bystander cells. Adapted from Szőke et al. (2012)

Distributions of transformed basal and secretory cells in a bronchial airway bifurcation model following exposure to radon progeny alpha particles for defined environmental and physiological inhalation conditions are plotted in Fig. 16 (Szőke et al. 2012). Since multiple hits are associated with the hot spot activity distribution, the highest oncogenic risk can be observed around the carinal ridge. Bystander mechanisms, such as cell-to-cell communication, disseminate damage to more distal cells, thereby enhancing the total oncogenic potential. Thus, these calculations demonstrate that the inhomogeneity of the radon progeny deposition pattern leads to an inhomogeneous energy deposition distribution, which in turn produces a non-uniform distribution of inactivation and transformation probabilities among the exposed cells.

Related to the concept of an average track length in a spherical target, Carlson et al. (2008) and Stewart et al. (2011) used the frequency-mean specific energy per radiation event, $\bar{z}_{\text{F}}$, as a function of LET and target diameter as the basis for subsequent radiobiological simulations for different ions, including alpha particles. By combining Monte Carlo DNA damage simulations with deterministic repair models, they examined putative mechanisms of cell killing (Carlson et al. 2008), specifically the effect of radiation quality and cellular oxygen concentration on clustered DNA lesions and related reproductive cell death.

### Models based on specific energy distributions

If specific energy distributions were calculated for given exposure conditions, the question arises how these specific energy distributions can be used to predict the radiobiological response. In other words, can these distributions in specific energy, or parts of them, directly be related to specific cellular radiation effects?

#### The theory of dual radiation action

The theory of dual radiation action is based on the assumption that the biological response to an irradiated cell results from the interaction of two sublesions in a sensitive volume (Kellerer and Rossi 1972, 1978). These sublesions are produced by the energy imparted to a spherical site and have a fixed probability of interacting pairwise. Since the number of sublesions is proportional to the specific energy *z* in the target volume, the expectation value for the number of resulting lesions is proportional to the expectation value of the square of the specific energy in that site:

10 $$ E\left( z \right) = kz^{2} $$

The mean number of lesions as a function of the absorbed dose *D*, *E*(*D*), can then be obtained by averaging over *E*(*z*) for all sensitive volumes:

11 $$ E\left( D \right) = \mathop \int \limits_{0}^{\infty } kz^{2}  f\left( {z;D} \right){\text{d}}z = k\bar{z}^{2} \left( D \right) $$

Since the expectation value z^2^ (*D*) of the square of *z* at dose *D* is:

12 $$ \bar{z}^{2} \left( D \right) = \xi D + D^{2} $$

where ζ is the average specific energy produced in individual events in the site, i.e., the single-event dose mean specific energy. This finally leads to a linear-quadratic dose–effect relation:

13 $$ E\left( D \right) = k\left( {\xi D + D^{2} } \right) $$

The average diameter of the sensitive volume over which sublesions are combined is typically a few micrometers. Although the very nature of the sublesions is not specifically defined, the production of DNA double-strand breaks as a result of the interaction of two adjacent single-strand breaks and the formation of chromosome aberrations from pairs of chromosome breaks possess the characteristics of dual radiation action. The interaction of sublesions can either be interpreted that two sublesions must be formed within a given site to interact (site model) or one can assume that the interaction is determined by the diffusion of sublesions within the cell over certain distances (distance model).

At a dose equal to ζ, the linear component in the dose–effect relation is equal to the quadratic component. The linear component (intratrack action) dominates for lower doses, while the quadratic component (intertrack action) dominates for larger doses. In the case of low doses of alpha particles, i.e., single alpha particle hits in irradiated cells, double-strand breaks are produced by the interaction of two single-strand breaks generated within the same alpha particle track, thus exhibiting a linear response. For comparison, intratrack action is characteristic of low-LET radiations.

In their generalized formulation of the dual radiation action, Kellerer and Rossi (1978) refined the concept of the dual radiation action, and the specific energy *ζ* in the linear term of the linear-quadratic dose–response relation is replaced by the function *ξ*. This function can be expressed by three functions, the proximity function of the sensitive matrix *s*(*x*), which describes the geometry of the sensitive material in the cell, the proximity function of the energy transfers *t*(*x*), which characterizes the geometry of the pattern of energy deposition within the sensitive matrix, and the interaction probability *g*(*x*), which defines the interaction probability of sublesions as a function of their separation. Provided that the geometry of the sensitive matrix and the microdistribution of energy transfers are known, the three functions allow the calculation of the yield *E*(*D*). Although the dual radiation action model is a useful tool for the interpretation of dose–effect curves, it does not allow the a priori prediction of specific biological endpoints.

#### Effect-specific threshold models

The concept of effect-specific thresholds is based on the assumption that specific energies below and above a defined threshold specific energy *z*_o_ can be related to specific radiobiological effects, such as cell killing or transformation (Fisher 1988; Fisher et al. 1992; Hui et al. 1990; Sedlák 1996).

Fisher (1988) correlated the probability of long-term radiobiological effects with specific energy distributions for seven different dose levels in beagle dog lungs following inhalation of ^239^PuO_2_ aerosols. Average cumulative lung doses were 0.25, 0.8, 1.5, 5, 12, 25 and 60 Gy, respectively. The comparison with lung cancer incidence levels suggested that specific energy distributions in the interval between 5 and 25 Gy may be related to the preferential occurrence of lung tumors, while early death due to radiation pneumonitis will dominate above a threshold dose of 40 Gy.

Hui et al. (1990) computed specific energy distributions in basal and secretory cells in all bronchial airway generations of the human lung for alpha particles emitted from inhaled radon progeny. Assuming that specific energies greater than 0.5 Gy to the cell nucleus are lethal, the risk associated with the exposure to radon progeny can be estimated by integrating the probability of specific energies less than 0.5 Gy.

It can be assumed that a cell receiving a specific energy below a threshold *z*_o_ may survive and continue division. Since the value of *z*_o_ is most likely not constant but a continuous function *s*(*z*), the probability for a biological endpoint for the surviving cells, E(S), is given by:

14 $$ E\left( S \right) = k\mathop \int \limits_{0}^{\infty } s\left( z \right)\lambda \left( z \right)f\left( {z;D} \right){\text{d}}z $$

where *k* is a proportionality constant and *λ*(*z*) is the probability of a cell being transformed (Fisher et al. 1992). The function *λ*(*z*) is also called hit-size effectiveness function (see “Hit-size-effectiveness function”).

Sedlák (1996) proposed the concept of a so-called boundary specific energy *z*_o_, which distinguishes between glancing cellular hits, which are non-lethal and possibly carcinogenic, and alpha particle traversals near the center of the nucleus, which probably inactivate the cell. Consequently, lung cancer frequency for inhaled radon progeny is related to the number of sensitive cells receiving glancing hits. Thus, for a given specific energy distribution *F*(*z*,*D*), if *z* > *z*_o_, then the inactivation of the cell is expected, while if *z* < *z*_o_, this may lead to an oncogenic event. The distribution function *F*(*z*_o_,*D*) represents the fraction of cells with specific energy ≤ *z*_o_ at dose *D*, which also includes the fraction of cells which are not hit, i.e., z = 0. Thus, the fraction of glancing hits $G\left( D \right) = F\left( {z_{0} ;D} \right) - F\left( {0;D} \right)$. The concept of the boundary specific energy might be generalized by assuming a distribution in *z*_o_ values instead of a single *z*_o_ value, resembling the concept of the hit-size effectiveness function (Bond et al. 1985; Fisher et al. 1992; Varma et al. 1994).

The concept of the boundary specific energy was tested against radiobiological in vitro experiments for cell killing (cellular survival) and oncogenic transformation (Sedlák 1996). Based on the analyses of survival and transformation curves with different radiations and cell lines, average *z*_o_ values of 0.44 Gy for cell killing and 0.43 Gy for the transformation frequencies per surviving cell could be derived. The similarity of both values suggests that a single *z*_o_ value may be sufficient for the microdosimetric interpretation of lung cancer risk.

#### Hit-size effectiveness function

The concept of the hit-size effectiveness function (HSEF) was introduced by Bond et al. (1985) to relate microdosimetry to different radiobiological endpoints. It provides a formal description of the dose–effect relationship without any assumptions on cellular mechanisms of radiation action. The HSEF concept combines the hit concept with the specific energy distribution concept. The basic assumption of this concept is that both energy deposition in subcellular sites and the subsequent cellular response are random processes. Hence, the dose–effect relationship *E*(*D*) was expressed by Morstin et al. (1989) as an integral convolution of two separate functions, the specific energy *f*(*z*), which describes energy deposition within a sensitive volume of irradiated cells, and the hit-size effectiveness function *ε*(*z*), which represents the cellular response to ionizing radiation in a specified way:

15 $$ E\left( D \right) = \mathop \int \limits_{0}^{\infty } f\left( z \right){\text{d}}z $$

For low level exposures of high-LET radiations, e.g., alpha particles, where it is unlikely that a cell is hit more than once, the number of cells hit increases with increasing exposure, but not the average energy deposited in those cells. Thus, *f*(*z*, *D*) can be replaced by the single-event distribution *f*_1_(*z*), and the cellular response function can be expressed as:

16 $$ \varepsilon \left( z \right) = kP\left( z \right) $$

where *P*(*z*) is the probability of a specified quantal response (“all-or-none” logic) to a hit of size *z*, and *k* is a proportionality constant. The relevant site size for the calculation of *f*_1_(*z*) distributions is typically of the order of a few micrometers. Since the site size hardly affects the derivation of the HSEF, a target critical volume (TCV) of 1 μm is commonly adopted.

The hit-size effectiveness function formally represents the cumulative probability that a subcellular target structure will respond to a specific target-averaged ionization density. Since it does not uniquely describe the radiation–target interaction, relative HSEF functions must be derived by fitting probability distributions to specific experimental radiobiological data, such as mutations, chromosome aberrations and inactivation, for low- and high-LET radiations (Morstin et al. 1989; Sondhaus et al. 1990; Varma et al. 1994). The value of the HSEF at each event size *z*, multiplied by the fraction of cells hit at that *z* value, and summed over all event sizes, yields a single value representing the fraction of quantally responding cells.

**Fig. 17:**
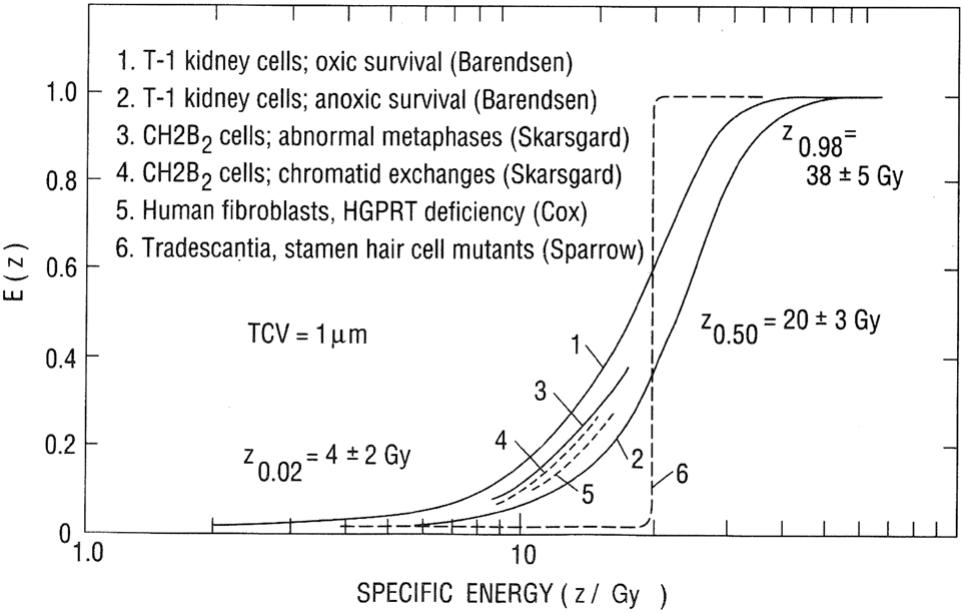
Hit-size effectiveness functions *E*(*z*) for six different biological endpoints derived from measurements with various types of radiations with LETs ranging from 1 to 350 keV μm^−1^. Specific energy distributions refer to a target critical volume (TCV) of 1 μm. Values of *E*(*z*) represent the probability of quantal response to a hit of size *z*, computed as the ratios of the relative number of responding cells per exposed cell to the relative number of hits per exposed cell, calculated for each *z* interval. Approximate values for which quantal response probabilities *E*(*z*) are 0.02, 0.50, and 0.98 are indicated on the graph. Reprinted from Sondhaus et al. (1990) with permission of Wolters Kluwer Health, Inc

Examples of HSEFs for a variety of biological endpoints derived from measurements in different cell systems and types of radiations are shown in Fig. 17 (Sondhaus et al. 1990), exhibiting a typical sigmoid shape (Bond et al. 1985). Values of *E*(*z*) represent the probability of a quantal response to a hit of size *z*, computed as the ratios of the relative number of responding cells per exposed cell to the relative number of hits per exposed cell, calculated for each *z* interval. The similarity of the HSEFs for the different cell responses suggests that a single hit-size effectiveness function might suffice for radiation protection purposes.

### Track structure models

#### Amorphous track structure model

In the amorphous track structure model of Katz (1987), radiobiological effects in cellular targets are related to the radial dose distribution around a charged particle track through four radiosensitivity parameters, which can be obtained from effect-specific in vitro studies (Katz 1987; Katz and Hofmann 1982; Katz et al. 1994). This model has been applied to the cellular radiation effects in basal and secretory cells of the bronchial epithelium irradiated by radon progeny alpha particles (Hofmann 1988; Hofmann and Daschil 1986; Hofmann et al. 1996c, 1985, 1986). Cellular radiosensitivity parameters applicable to the prediction of carcinogenic response were derived from in vitro data on oncogenic transformation and survival of C3H 10T1/2 mouse embryo fibroblast cells (Hei et al. 1988; Waligorski 1990), HPRT mutation and survival in V79 Chinese hamster cells (Thacker et al. 1979; Hofmann and Daschil 1985), and chromatid aberrations and survival in CH2B_2_ cells (Skarsgard et al. 1967; Waligorski 1990). Energy spectra of ^218^Po and ^214^Po alpha particles were computed for cell nuclei located at varying depths in the bronchial epithelium of different airway generations (Caswell et al. 1994). Based on these alpha particle energy spectra and the track structure formalism, the number of observable inactivations, chromatid aberrations, oncogenic transformations and mutations were calculated for selected airway generations, weighted by the depth density distributions of basal and secretory cell nuclei. In the framework of the initiation–promotion concept of radiation carcinogenesis, mutation and transformation may be interpreted as initiating events, while inactivation or chromosome aberrations may be interpreted as indicators of the promotional effect of cell killing (Crawford-Brown and Hofmann 1990).

A similar modeling approach was presented by Elsässer and Scholz (2007) and Friedrich et al. (2012) by relating experimental data of DNA damages produced by ions and photons to the radial dose profile of the amorphous track structure model. This local effect model (LEM) describes the spatial distribution of initial DNA damages, e.g., single- or double-strand breaks, produced by heavy charged particles used in radiotherapy, although not specifically for alpha particles. Such calculations revealed that clusters of DNA lesions at the nanometer and micrometer scale lead to an enhanced severity of induced cellular damage, e.g., cellular inactivation or induction of mutations (Friedrich et al. 2018).

#### Nanoscale track structure model

Nanodosimetry or nanoscale dosimetry is concerned with the calculation of the track structure of charged particles down to nanoscale resolution. At this scale, which is comparable to the dimensions of DNA base pairs, energy deposition is no longer the result of a large number of ionizations, but is characterized by the formation of ionization clusters within nanometer-sized target volumes (Rabus and Nettelbeck 2011; Palmans et al. 2015). Since a comprehensive description of track structure simulations has already been presented in “Alpha particle tracks in silico: from nanoscale to microscale”, only a few examples of track structure calculations for the prediction of DNA damages, such as double-strand breaks, are listed here.

The first “ab initio” mechanistic approach to radiation-induced chromosomal aberrations and their kinetics, combining track structure simulations with detailed models of chromatin structure and accounting for the kinetics of DNA repair has recently been reported by Friedland and Kundrát (2013, 2015) and Friedland et al. (2010) for gamma rays and alpha particles. Track structure calculations, performed with the Monte Carlo track structure code PARTRAC (see “Alpha particle tracks in silico: from nanoscale to microscale”), provide the starting conditions for the subsequent biological response models. The initial DNA damages are computed by overlapping space- and time-dependent track structures with multi-scale DNA and chromatin models, ranging from DNA double-helix in atomic resolution to chromatin fiber loops, heterochromatic and euchromatic regions, and chromosome territories. These calculations provide information on the spatial distribution of double-strand breaks (DSBs) and their complexity.

Other examples of track structure calculations are the prediction of DSBs produced by electrons, protons and alpha particles has (see Fig. 14) and the formation of dicentrics (chromosome exchange aberrations with two centromeres) after gamma and alpha irradiation in human fibroblasts (Friedland and Kundrát 2013, 2015) and their comparison with the experimental data of Conforth et al. (2002).

## From cellular microdosimetry to the tissue and organ level

Calculated microdosimetric parameters, such as specific energy distributions, hit probabilities or track structure simulations discussed in previous sections refer to energy deposition in single cell nuclei. Hence, the question arises whether energy deposition in specific cells characterized by microdosimetric quantities can be correlated with radiobiological effects at the tissue or organ level, such as epidemiologically observed cancer risk.

To understand health effects of alpha radiation at the tissue or organ level, it is imperative to understand how physical effects of ionizing radiation at the microscopic scale propagate over different levels of biological organization. While it is crucial in carcinogenesis how cellular effects manifest themselves at the tissue level, experimental evidence points to the microenvironment and the tissue level as critical targets for late effects, and to epigenetic and signaling mechanisms as mediators of radiation damage (Barcellos-Hoff and Brooks 2001; Barcellos-Hoff et al. 2014). This higher structural level is generally ignored in modeling due to its complexity and the lack of experimental data.

The application of microdosimetric quantities to the analyses or predictions of cancer risk is limited by two major factors. First, carcinogenesis is a multicellular event invoking the interaction of cells surrounding the cell receiving an energy deposition event. For example, the histogenesis of bone sarcoma after internal irradiation with alpha-emitters shows that the final histopathology of deterministic or stochastic endpoints depends on the microenvironment of a target cell, which has to be regarded as a synergistic morpho-functional tissue unit (“histion”) (Goessner 2003). Furthermore, several non-targeted effects have been reported in the literature where cells hit by alpha particles induce a radiobiological response in non-irradiated neighboring cells (Sawant et al. 2001; Fakir et al. 2009; Belchior et al. 2014; Belchior et al. 2013). Non-targeted mechanisms comprise detrimental as well as protective bystander effects, genomic instability, adaptive response, induction of apoptosis and repopulation by cell killing (Truta-Popa et al. 2011b).

Second, carcinogenesis is a multi-step process consisting of a sequence of initiating, such as oncogenic transformation, and promotional mechanisms, such as radiation-induced proliferation or cigarette smoking as in the case of lung cancer (Truta-Popa et al. 2011b). Hence, the microdosimetric parameters referring to energy deposition at a given point in time cannot account for modifications of the initial response as a function of time after exposure. A special case is the low dose and dose rate exposure, where alpha particle hits in a given cell nucleus are widely spaced in time and a given cell hit once may have already been replaced by another cell during subsequent cell cycles at the time of another hit (Chadwick et al. 2003).

Thus, to consider the above effects, specific energy distributions or hit probabilities must be supplemented by additional information on the tissue-specific biological mechanisms described above. A first simplified approach is the direct application of specific energy distributions to published cancer data by deriving specific energy thresholds from these data as described by the threshold models presented in “Alpha particle tracks in silico: from nanoscale to microscale” (Fisher 1988; Fisher et al. 1992; Hui et al. 1990; Sedlák 1996). For example, specific energy distributions in the interval between 5 and 25 Gy may be related to the preferential occurrence of lung tumors in beagle dog lungs following inhalation of ^239^PuO_2_ aerosols, while early death due to radiation pneumonitis will dominate above a threshold dose of 40 Gy (Fisher 1988). Likewise, Sedlák (1996) proposed that lung cancer frequency for inhaled radon progeny is related to the number of sensitive cells with glancing hits which are non-lethal and possibly carcinogenic, suggesting a threshold dose of about 0.4 Gy in sensitive bronchial target cells. While these thresholds provide lower and upper limits of doses for the occurrence of lung cancers, they do not allow the prediction of cancer cases as a function of cumulative exposure.

A second approach has been based on the assumption that cancer risk is related to the number of transformed cells derived from in vitro experiments (see “Relationship between microdosimetry and cellular radiobiological effects”), either related to specific energy distributions or hit probabilities, and supplemented by functions describing the concomitant response to cellular inactivation (Truta-Popa et al. 2011a) or the contribution of non-targeted mechanisms (Truta-Popa et al. 2011b).

In the framework of the transformation frequency–tissue response (TF–TR) model (Truta-Popa et al. 2011a), lung cancer risk *R*(*D*) resulting from inhaled radon progeny is expressed as the product of the transformation frequency function TF(*D*) and the tissue response function TR(*D*), based on experimentally observed cellular transformation and survival studies. While oncogenic transformation is assumed to represent the primary initiation step, stimulated mitosis by killing adjacent cells, relating in vitro transformation in single cells to in vivo transformation in tissue, is interpreted as the primary tissue response mechanism. Average doses throughout defined exposure periods are expressed as the product of single and multiple hits within a given cell cycle time, selected from a Poisson distribution, the number of cell cycles within a given exposure period, and the average cellular dose per single hit. The fair agreement between model predictions and data on the relative lung cancer risk in US uranium miners (Hornung and Meinhardt 1987) suggests that in vitro oncogenic transformation data supplemented by a tissue response function represent a useful tool for lung cancer risk modeling. Furthermore, the relation of alpha particle hits to the cell cycle time of an irradiated cell leads to an inverse dose rate effect which has been observed in epidemiological lung cancer (Lubin et al. 1995) and radon rat inhalation studies (Monchaux 2004).

To investigate the shape of lung cancer risk function at chronic, low level radon exposures in more detail, non-targeted mechanisms operating specifically at low doses were incorporated into the TF–TR model (Truta-Popa et al. 2011a). While genomic instability and bystander effects amplify the biological effectiveness of a given dose, induction of apoptosis and adaptive response will reduce the carcinogenic risk. Predictions of lung cancer risk including these non-targeted mechanisms exhibit a distinct sublinear dose–response relationship at low exposures, particularly for very low exposure rates.

Based on the amorphous track structure model of Katz (1987), epidemiological data on lung cancer incidence in a Chinese high background area arising from the inhalation of ^222^Rn and ^220^Rn progeny were analyzed by an incidence function considering cell killing as competitive with malignant transformation (Hofmann et al. 1986). Track structure predictions for specific bronchial airway generations suggested that lung cancer risk per unit exposure of inhaled ^222^Rn radon progeny in uranium miners is either constant or increases slightly at low exposure levels up to a cumulative exposure of about 100 WLM, and then decreases significantly at high cumulative exposures (Hofmann et al. 1986), consistent with the epidemiological evidence.

Based on a microdosimetric description of the interaction of radon progeny alpha particles and a numerical model of the cellular structure of the bronchial epithelium, Madas et al. (2011), Madas and Balásházy (2011) and Madas and Varga (2014) investigated the role of cell killing on mutation frequencies in tissue, arising from misrepaired DNA damages and eventually leading to mutations and, in further consequence, to carcinogenesis. Cellular hit distributions were determined for different cell types in both the deposition hot spots at bronchial airway bifurcations (Madas and Balásházy 2011) and in parts of the large bronchi (Madas et al. 2011). Since alpha particles effectively kill cells, the replacement of killed cells also contributes to mutagenesis as spontaneous mutations occur during cell division. While the increase of the cell division rate and the induction of DNA damage exhibit a synergistic response, results indicated that radon progeny alpha particles primarily cause mutations in the epithelium via the induced replacement of killed cells (Madas and Balásházy 2011).

Madas (2016), Madas and Drozsdik (2018), and Drozsdik and Madas (2019) also explored the role of hyperplasia on radon-induced lung cancer risk. The conjecture that hyperplasia occurs in the highly exposed carinal regions of the bronchial airways is supported by experimental (Rogers 2003) and histological findings (Auerbach et al. 1961). Simulations indicated that an increase in progenitor cell number reduces not only the cell division rate for a given exposure rate, but also the microscopic energy deposition in the tissue (Madas 2016; Madas and Drozsdik 2018). As the number of progenitor cells increases, the bronchial epithelium tissue becomes thicker, thereby increasing the average distance between alpha decay sites and progenitor cells, and hence reducing the mean number of cellular hits. Therefore, the induction of hyperplasia in the bronchial epithelium can be considered as an adaptive response of the tissue to chronic radon exposure (Madas 2016). The induction of hyperplasia also provides a potential explanation for the inverse exposure rate effect (Madas and Balásházy 2011) observed in epidemiological studies (Lubin et al. 1995) and the dependence of clonal expansion rate on exposure rate observed in mathematical analyses of epidemiological data (Zaballa and Eidemüller 2016).

As opposed to the above approaches, which are based on microdosimetric predictions of oncogenic transformation and mutation frequencies modified by cell killing and concomitant biological mechanisms, a third approach follows the opposite direction, i.e., the incorporation of microdosimetric parameters into carcinogenesis models (Madas and Varga 2014; Fakir and Hofmann 2006; Drozsdik and Madas 2019).

In the generalized state-vector model of radiation carcinogenesis (SVM), it is assumed that a cell must pass through a sequence of several initiation and promotion stages to produce the related carcinogenic effects (Crawford-Brown and Hofmann 1990, 2002). Transition rates of the initiation phase are expressed as functions of microdosimetric parameters by describing the complexity and clustering of DNA double-strand breaks (Fakir et al. 2006) and their effect on repair kinetics, while the promotion phase is based on a multi-target single-hit formulation of damages to specific targets within the cell which are important for preserving contact inhibition (Fakir and Hofmann 2006). Such an approach allows the consideration of hit frequencies and the variability of specific energy spectra of radon progeny alpha particles in bronchial target cells for defined exposure conditions.

The analysis of epidemiological data with the two-stage clonal expansion (TSCE) model (Moolgavkar and Luebeck 1990) requires mathematical functions describing the dose dependence of mutation induction (for initiation and transformation) and clonal expansion (promotion). These functions are presently derived from radiobiological in vitro experiments with different cell lines. However, considering only single cell responses may be misleading, as high doses result in cell killing with high probability without producing any mutations at the single cell level, while mutations do occur at the tissue level due to the replacement of killed cells. Thus, microdosimetric parameters together with biological response models at the tissue level can provide more realistic functions to be applied in the TSCE model or other models used for the analysis of epidemiological data (Madas and Varga 2014).

In conclusion, the above discussion on a potential correlation between microdosimetric quantities and carcinogenic risk observed in epidemiological studies or animal experiments clearly demonstrates the limited applicability of specific energy distributions and hit probabilities in individual cell nuclei eventually leading to oncogenic transformation. Indeed, due to the complexity of the carcinogenic process, biological mechanisms seem to play a much greater role than the initial energy deposition event. Thus, microdosimetric parameters, such as specific energy distributions or hit probabilities, though providing useful information at the beginning of the chain of biological mechanisms, will be of limited relevance for radiation protection purposes. Note, however, that cell killing is the dominating interaction of alpha particles with cell nuclei. Thus while this limitation of energy deposition in specific cells represents only the initiating step for the development of cancers, it does provide the necessary information on cell killing for the radiotherapeutic application of alpha-emitting radiopharmaceuticals (see “Alpha particle microdosimetry in targeted radionuclide therapy”). Therefore, microdosimetric quantities, such as specific energy distributions will be of limited use for radiation-induced carcinogenesis, but represents a useful and important tool in therapy with alpha-emitting radionuclides.

## Applicability and limitations of alpha particle microdosimetry

“Classical microdosimetry” deals with the determination of probability densities of “specific energy”, as measured by proportional counters, and related statistical quantities in microscopic volumes and attempts to correlate these specific energy densities directly with experimentally reported biological radiation effects through the dual radiation action hypothesis (Kellerer and Rossi 1972). However, the concentrations of energy and activations at three structural levels, i.e., in atoms (1 Å), macromolecules (10 nm) and cell nuclei (1 μm) lead to deviations from proportionality between specific energy and biological changes (Paretzke 1978). Thus, specific energy spectra in micrometer regions cannot account for details in charged particle track structures and their resulting biological effects, because energy density is measured in large volumes instead of the pattern of activations on a much smaller scale. This implies that track structure calculations are required to fully explore the relationship between the spatial structures of charged particle tracks and experimentally reported biological endpoints. These limitations, however, do not question the concept of specific energy per se but rather its relevance for predicting biological effects originating in nanometer regions. Indeed, as discussed in “Relationship between microdosimetry and cellular radiobiological effects”, the attempt to establish a relationship between specific energy spectra and specific cellular biological effects is presently still an unresolved issue. This can only be accomplished by future track structure calculations.

Regarding the prediction of biological effects, microdosimetry describes only the initial step of energy deposition in cellular sites, but not subsequently operating biological mechanisms, such as DNA repair or intercellular communication. Thus, microdosimetric concepts are most successful in exposure situations where the biological effect is dominated by energy deposition events, such as DNA strand breaks or cell killing (cellular survival), but less successful in cases where the final biological effect is dominated by subsequently acting biological factors, such as chemical radical attacks and repair mechanisms.

The application of microdosimetric quantities to the analyses of cellular radiobiological effects or predictions of cancer risk is limited by two major factors. First of all, carcinogenesis is a multicellular event invoking the interaction of cells surrounding the cell receiving an energy deposition. Furthermore, several non-targeted effects, such as genomic instability, adaptive response, induction of apoptosis and repopulation by cell killing, have been reported in the literature where cells hit by alpha particles induce a radiobiological response in unirradiated neighboring cells. Hence, the microdosimetric parameters referring to energy deposition in single cells cannot account for the interaction of cells within a given tissue volume or between hit and non-hit cells as described by these non-targeted mechanisms. Second, carcinogenesis is a multi-step process consisting of a sequence of initiating, such as oncogenic transformation, and promotional mechanisms, such as radiation-induced proliferation. Hence, the microdosimetric parameters referring to energy deposition at a given point in time cannot account for modifications of the initial response as a function of time after exposure.

The most important biological endpoint of alpha particle exposure in radiation protection is cancer induction, such as the occurrence of bronchial cancers initiated by alpha particle irradiation of bronchial target cells resulting from the inhalation of radon progeny. However, the discussion in “Relationship between microdosimetry and cellular radiobiological effects” and “From cellular microdosimetry to the tissue and organ level” on the potential correlation between microdosimetric quantities and carcinogenic risk observed in epidemiological studies or animal experiments clearly demonstrated the limited applicability of specific energy distributions and hit probabilities in individual cell nuclei eventually leading to oncogenic transformation and carcinogenesis. On the other hand, microdosimetry can provide some guidance on the shape of dose–effect curves, such as the linear relationship at low doses or the reduction of the carcinogenic response at high doses. Furthermore, microdosimetry may also help to understand some basic cellular mechanisms eventually leading to cancer in the framework of carcinogenesis models, such as clonal expansion models. Therefore, the calculation of microdosimetric parameters in radon lung cancer risk assessment may help to improve the knowledge at different levels of arguments, listed below:

1. Low doses delivered by radon progeny alpha particles to lung tissue are characterized by a small number of cells affected as opposed to the common view in macroscopic dosimetry that all cells in the tissue receive the same average dose. At low dose rates, isolated cellular energy deposition events are spaced in time over an extended period of time. Thus, macroscopic doses in the low dose range ought to be replaced by corresponding microdosimetric distributions. At the lowest possible dose, i.e., delivered by a single alpha particle, the question arises whether a single alpha particle can give rise to a tumor, thus questioning the applicability of the LNT hypothesis.
2. Another issue is the value of the radiation weighting factor, w_R_, for alpha particles which is presently set at 20 (ICRP 2003) relative to 1 for low-LET radiations. While *w*_R_ values represent judgements based on maximum RBE values, measured RBE values for in vitro oncogenic transformation in different cell lines, irradiated by alpha particles with varying LET, ranged from 3 to 10, i.e., considerably lower than the current value of 20 (Hofmann et al. 2004). Note that RBE is defined as the ratio of a specific biological effect per unit dose for high-LET radiations to that same effect per unit dose for low-LET radiations. Since specific energy distributions for gamma and alpha irradiation are quite different, this comparison of effects ought to be based on microdosimetric distributions rather than on macroscopic doses.
3. Apportionment factors for the bronchial and the alveolar region, which represent the relative contributions of each lung region to lung cancer risk, are currently applied to average bronchial and alveolar doses. While a strong geometric correlation exists in bronchial airways between the cylindrical alpha particle source and cellular target sites, both alpha particle emission and target sites are randomly distributed in the alveolar region. As a result, specific energy distribution for the same average macroscopic dose differs considerably from each other (Hofmann et al. 1990a; Fakir et al. 2005b, 2006). Thus, the values of these apportionment factors should by based on specific energy distributions instead of average bronchial and alveolar doses.
4. Two different approaches are currently used by ICRP (2010) to assess lung cancer risk due to radon inhalation: the epidemiological and the dosimetric approaches. The epidemiological approach is based on the ratio of lung cancer risk per unit exposure to radon progeny in miners and the nominal risk per unit effective dose of all cancers derived primarily from atomic bomb survivors who were exposed largely to gamma rays. In the dosimetric approach, macroscopic equivalent doses to organs and effective doses are calculated for defined radon progeny aerosol properties and breathing conditions using dosimetric models. Differences in specific energy distributions between gamma radiation and alpha particles affect the ratio of unit effective dose and unit radon exposure in the epidemiological approach and, in further consequence, the agreement between the epidemiological and the dosimetric approach.

The microdosimetric approach is especially relevant for the dose–response relationship at low doses and dose rates, where alpha particle hits in a given cell nucleus are widely distributed in space and in time and a given cell hit once may have already been repaired or replaced by another cell during subsequent cell cycles prior to a second hit. Thus, biological effects at low doses and dose rates are related to the interaction of single alpha particles within cell nuclei. Since energy deposition by single hits is accurately described by single-hit probabilities or single-event distributions, low dose effects may be appropriately characterized by microdosimetric parameters.

Regarding radiotherapy with alpha-emitting radionuclides, it is important to note that cell killing is the dominating biological effect of alpha particle interactions with cell nuclei. Since cell killing is the most relevant biological endpoint for the radiotherapeutic application of alpha particles, microdosimetry does provide the necessary information on cell killing for the application of alpha-emitting radiopharmaceuticals (see “Alpha particle microdosimetry in targeted radionuclide therapy”). Thus, microdosimetric quantities, such as specific energy distributions will be of limited use for radiation-induced carcinogenesis, but represents a useful and important tool in radiotherapy with alpha-emitting radionuclides.

The theory and the techniques of the microdosimetric approach have been well established. However, there is a gap in research application of biokinetic models at the microscopic level for the emerging targeted alpha radiopharmaceuticals, since molecular imaging has quickly developed and can provide the distribution information of radionuclides at the cellular and subcellular levels.

Microdosimetry is commonly regarded as a bridge between macrodosimetry, such as absorbed organ dose, and nanodosimetry, such as the dose to DNA moiety or to mitochondria, and the description of energy deposition other than the concept of dose. If we want to establish a relationship from initial tracks, energy deposition and chemical radicals at the molecular level to the tissue or organ level, microdosimetry is an inevitable intermediate. By upgrading to average absorbed dose, for example, lineal energy can be implemented in radiation quality formulation which is still important in radiation protection; *y* or *z* instead of LET and absorbed dose can be used in cellular survival fractions to explain the biological mechanism effects at a cellular level; *f*(*y*) and *f*(*z*) can be applied in mechanistic radiation mechanisms modeling. By downgrading to nanodosimetry, for example, determination of the microscopic quantities *y* and *z* needs a detailed spatial and temporal distribution of physical energy deposition or chemical radical attack, and even the biological effects, which can only be evaluated by nanodosimetric track structure calculations. There is still incomplete knowledge of the required techniques and hence new methods need to be developed, such as the microdosimetric formulation of NTCP (normal tissue complication probability) and TCP (tumor control probability) in alpha radiopharmaceutical therapy.

In the framework of the European Radiation Dosimetry Group (EURADOS), two working groups are collaborating closely in the research field of micro- and nanodosimetry: the “Nanodosimetry Task Group” in Working Group 6 (WG6)—“Computational Dosimetry” and the “Internal Microdosimetry Task Group” in WG7—“Internal Dosimetry”. Both groups have the vision to sustain competence and training in experimental and theoretical microdosimetry and nanodosimetry research across Europa. Furthermore, WG6 is aiming to develop computational methods for the simulation of the initial events and the chemical stages of alpha-emitters. On the other hand, WG7 is focusing on developing mathematical models and combining the molecular initial events with the absorbed dose level. EURADOS, as an entity, can further cooperate with other organizations, such as ICRP, ICRU, EANM and MIRD for joint challenging projects, such as low dose risk estimation.

In conclusion, two different pathways can be envisioned for the future application of microdosimetric methods in internal alpha particle microdosimetry. Since microdosimetry characterizes only the initial step of energy deposition, microdosimetric concepts are most successful in exposure situations where biological effects are dominated by energy deposition, which eventually may require the application of track structure models at the nanometer scale. On the other hand, with increasing spatial scale, initial effects of energy deposition may be modified by subsequently operating biological mechanisms, which eventually may require the application of biological models at the tissue level.

## Footnotes

1 The Dirac delta function is a function equal to zero everywhere except for zero and whose integral is equal to one.

2 Working level month (WLM) is a historical unit of potential alpha energy exposure (1WLM = 3.54 mJ h m^−3^). It corresponds to the cumulative exposure of 1 working level (WL) for a working month of 170 h, where 1 WL is defined as any combination of short-lived radon progeny in 1 l air that will result in the emission of 1.30 × 10^5^ MeV of potential alpha energy (ICRU 2015).
